# From motion to molecules: exercise-triggered large EVs suppress oncogenic signaling in lung adenocarcinoma cells – an in vitro pilot study

**DOI:** 10.1186/s12935-026-04429-6

**Published:** 2026-07-29

**Authors:** Zoltan Adam, Kitti Garai, Abigel Sebok-Tornai, Marta Wilhelm, Krisztian Kvell

**Affiliations:** 1 Department of Pharmaceutical Biotechnology, Faculty of Pharmacy, University of Pécs, Pécs, Hungary; 2 Histology and Light Microscopy core facility at the Szentágothai Research Centre, University of Pécs, Pécs, Hungary; 3 Department of Laboratory Medicine, Medical School, University of Pécs, Pécs, Hungary; 4 Faculty of Science, Institute of Sport Sciences and Physical Education, University of Pécs, Pécs, Hungary

**Keywords:** Large extracellular vesicles (L-EVs), Physical exercise, Lung adenocarcinoma, MicroRNA (miRNA), Gene expression profiling, Tumor suppressive signaling

## Abstract

**Background:**

Although regular physical activity is known to reduce cancer risk, the circulating molecular mechanisms responsible for these effects remain incompletely defined. Exercise alters the release and composition of extracellular vesicles (EVs), suggesting a role in systemic anti-tumor signaling. However, the contribution of large EVs (L-EVs) ─ a distinct and understudied EV subtype ─ to the regulation of lung adenocarcinoma cell behaviour has not been examined to date.

**Methods:**

L-EVs were isolated from platelet-free plasma of healthy, regularly exercising older adults with over 25 years of training history (aged 61 ± 2 years), collected at rest (Pre) and after (Post) an acute endurance exercise bout. EV fractions were characterized via transmission electron microscopy (TEM), nanoparticle tracking analysis (NTA), and antibody-based profiling. PC9 lung adenocarcinoma cells were incubated with plasma-derived L-EV–enriched fractions for 24 h. Cell viability and metabolic activity were evaluated using ATP-based luminescence and XTT assays following L-EV treatment. miRNA and gene expression changes were assessed using RT-qPCR with TaqMan arrays. Differentially expressed miRNAs (DEmiRs) and genes (DEGs) were analysed using Ingenuity Pathway Analysis (IPA) to evaluate regulatory networks and predict functional outcomes.

**Results:**

Exercise significantly increased plasma nanoparticle concentration (mean increase of 5.05 × 10⁷ ± 9.99 × 10⁶ particles/mL; *p* < 0.001, Cohen’s d = 1.685). TEM and NTA confirmed successful isolation of L-EVs, with mode sizes of 136.1 ± 23.6 nm pre-exercise and 129.5 ± 17.7 nm post-exercise. In PC9 cells, both Pre- and Post-L-EVs reduced metabolic activity compared to control conditions, as demonstrated by ATP-based and XTT assays, indicating a functional impact on cell viability (*p* < 0.001). Transcriptomic analysis revealed distinct expression changes between conditions. Post-L-EVs downregulated oncogenic and immune evasion–associated genes (MAX, FASLG, and ELK1) while upregulating pro-apoptotic and cell cycle inhibitory genes (CASP9, FADD, and CDKN2B). Several DEmiRs, including miR-21-5p, miR-301b-3p, and miR-193a-3p, shifted in directions opposite to their known oncogenic patterns in NSCLC, suggesting a tumor-suppressive influence. IPA predicted enhanced apoptotic signaling and reduced metastatic potential following Post-L-EV treatment.

**Conclusion:**

Exercise-conditioned plasma-derived enriched L-EVs modulate gene and miRNA expression in lung adenocarcinoma cells toward a tumor-suppressive profile. By attenuating oncogenic and immune-evasion signals while enhancing apoptotic and cell cycle–inhibitory pathways, Post-L-EVs offer preliminary mechanistic insight into the anti-cancer potential of physical activity. Their origin from older adults engaged in long-term exercise highlights the systemic benefits of sustained activity and the promise of L-EVs as biomarkers of exercise responsiveness.

**Supplementary Information:**

The online version contains supplementary material available at https://doi.org/10.1186/s12935-026-04429-6.

## Introduction

Lung cancer stands as the most common malignancy and the primary cause of cancer-related mortality globally, whereas lung adenocarcinoma represents its most prevalent subtype [1, 2]. The late-stage diagnosis of most patients makes non-pharmacological prevention strategies and complementary care approaches more important than ever before.

Physical exercise postpones age-related physiological decline and lowers cancer risk [3–6]. Although exercise enhances cardiorespiratory and metabolic function [7], accumulating evidence shows that its strong cancer-preventive effects arise largely from circulating molecular mediators rather than from local physiological adaptations alone.

Among these factors, extracellular vesicles (EVs) have emerged as key mediators of interorgan communication [8]. Exosomes (30–150 nm) a subset of small EVs (sEVs) and microvesicles (100–1000 nm) a subset of large EVs (L-EVs) circulate in biofluids such as plasma, urine, and saliva and transport proteins, lipids, and non-coding RNAs to distant tissues [9–12]. Increasing evidence shows that exercise acutely elevates circulating EV levels, underscoring their potential role in systemic exercise adaptation [7, 13].

As a subset of EV biomolecular cargo, small non-coding RNA species, microRNAs (miRNAs) exert potent post-transcriptional regulatory effects on pathways governing proliferation, apoptosis, metabolism, immune responses, and angiogenesis [14–17]. Importantly, EV-delivered miRNAs affect disease progression, including cancer development and metastasis [18]. The cancer-preventive effects of physical exercise are well documented, yet the exact underlying molecular mechanisms require further elucidation [4, 19]. Research has shown that serum and plasma samples obtained immediately after an acute bout or following long-term exercise demonstrate anti-tumorigenic properties, including reduced cancer cell viability and increased apoptosis [20–24]. In their review, Papadopetraki and co-workers [25] emphasized that exercise responsive myokines and miRNAs released and packaged in vesicles in response to exercise may play a role in these tumor-modulatory effects. Previous studies have primarily examined changes in vesicle-free circulating miRNA levels in response to acute exercise [26–28] or changes in distribution of sEV fractions following acute exercise in healthy young men [29–32]. Studies of EV distribution and miRNA profiles in senior athletes as a result of long-term physical activity have received less attention [7, 33].

Early work investigating exercise-induced EVs in cancer models has focused almost exclusively on sEV -enriched preparations. Two recent reviews [34, 35] highlighted that experimental evidence in this field remains extremely limited, identifying only one preclinical study available at the time: Sadovska et al. [36], which used ultracentrifugation-derived sEVs and demonstrated reduced tumor growth and metastasis in a murine prostate cancer model without assessing miRNA or gene-expression changes. A subsequent study by Mlynska et al. [37] likewise utilized sEV isolates in a triple-negative breast cancer murine model, reporting tumor-suppressive and immunomodulatory effects, although only a limited set of immune-related genes was analyzed. Importantly, none of these studies examined L-EVs, a biologically distinct EV subtype formed by plasma membrane shedding and characterized by larger size, broader cargo coverage, and distinct biogenesis pathways [38]. As noted in these reviews, studies specifically addressing exercise-induced L-EVs are virtually absent, underscoring a critical gap in our understanding of EV subtype–specific contributions to exercise–cancer interactions.

Given the differences in EV biogenesis, cargo selection, and target cell interactions, it is essential to study distinct EV subtypes in the context of physical activity and disease [39]. In this study, we investigated whether an acute bout of exercise modifies the size distribution, concentration and cargo of human plasma-derived L-EVs in a way that influences human lung cancer cell behaviour. Using plasma samples collected before and after exercise from healthy adults (aged 61 ± 2 years) engaged in regular physical activity in the past 25 + years prior to the study, we assessed the impact of L-EVs on PC9 lung adenocarcinoma cells, focusing on miRNA and gene-expression changes. We hypothesized that exercise-conditioned large EVs contain regulatory miRNAs that contribute to systemic anti-tumor responses and thereby play a role in cancer prevention.

## Materials and methods

### Participants

A total of 40 senior adults were initially screened for inclusion through medical history, physical examination, and laboratory tests. Participants were recruited for sampling from a population of older adults who regularly engaged in endurance-type physical activity, via local sports clubs and university-affiliated exercise programs. Participation was voluntary, and individuals expressing interest were screened for eligibility based on predefined inclusion and exclusion criteria. Of these, 25 volunteers underwent full eligibility assessment based on predefined health, medication, and exercise-related criteria (see Additional file 1 and Fig.S1 for further detail). Eligible participants were healthy, over 60 years of age, and had engaged in regular physical activity for the past 25 years or more. Physical activity levels and lifestyle habits were assessed via a general lifestyle questionnaire and the International Physical Activity Questionnaire (IPAQ) [40]. Participants of both sexes were initially recruited; however, due to strict health- and safety-related inclusion criteria and the absence of female participants meeting all study requirements after full eligibility assessment, only male participants were included in the final analysis. Nine male participants (mean age: 61 ± 2 years) met the inclusion criteria and were enrolled. Details on participant characteristics, exercise history and body composition assessment are provided in the Supplementary Materials (see Additional file 1 and 2 for further detail). Each participant provided written informed consent before completing any data collection procedures. All subject recruitment, protocols and procedures described in this study were performed according to the Declaration of Helsinki principles and approved by the Regional and Local Ethics Committee of the Clinical Centre, University of Pécs (Ethics approval number: 7755-PTE 2019.).

### Study design

**Fig. 1:**
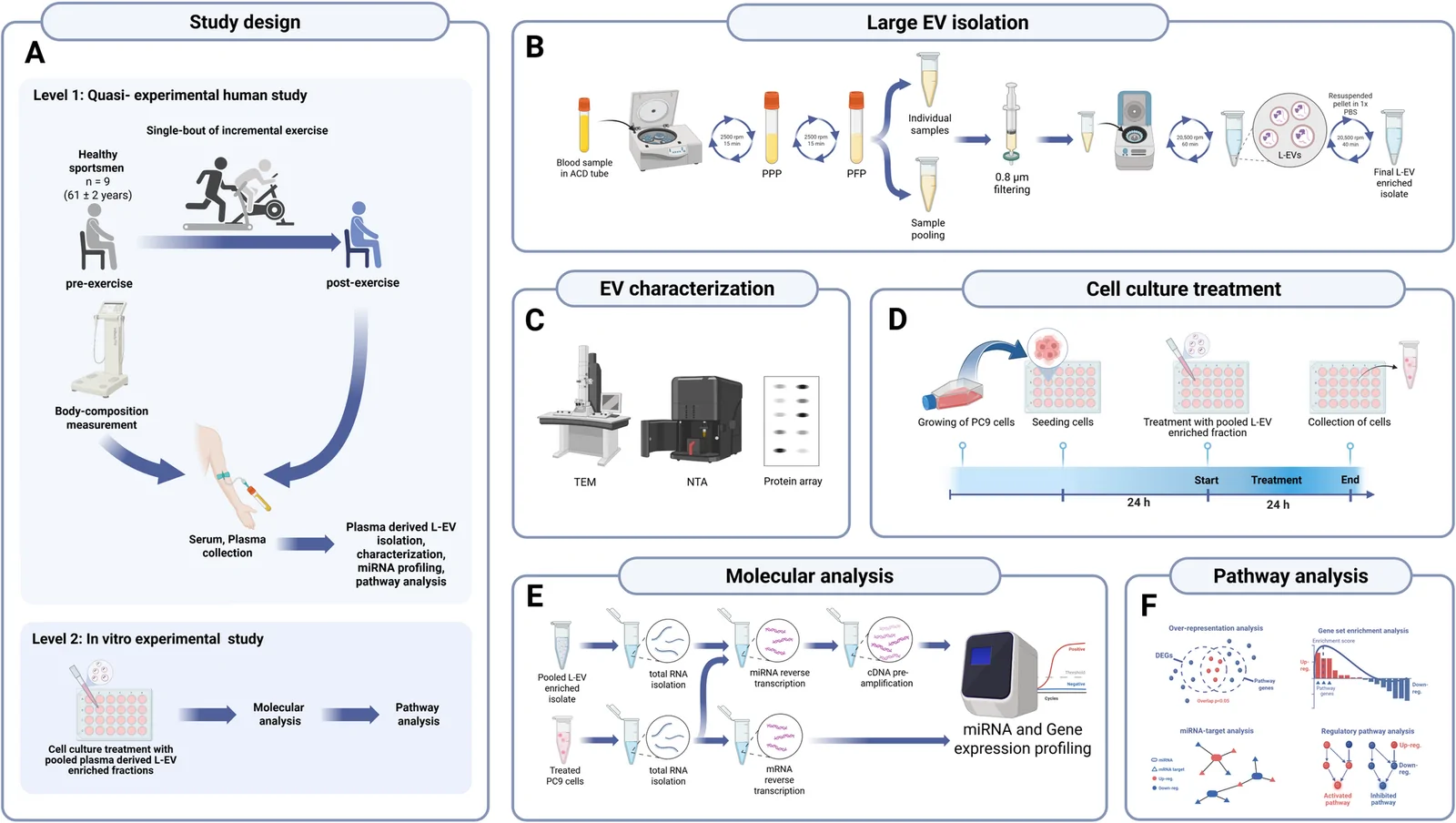
Experimental design and analytical workflow of the two-level study combining a quasi-experimental human pre-post exercise component and an in vitro experimental component. (**A**) Overview of the two-level study design. Level 1 (quasi-experimental human study): older, physically active participants (age: 61 ± 2 years; ≥ 25 years of regular physical activity) were recruited and subjected to laboratory assessments. Following baseline measurements (anthropometric assessment, body composition analysis, and fasting blood collection), participants completed a single bout of maximal exercise testing (VO₂max), followed by post-exercise blood sampling, L-EV isolation and characterization. Level 2 (in vitro experimental study): pooled large extracellular vesicle (L-EV)- enriched fractions derived from pre- and post-exercise plasma samples were applied as treatments to PC9 lung adenocarcinoma cells. (**B**) Isolation of L-EV enriched fraction from platelet-free plasma, a shared methodological step linking the human and in vitro study levels. (**C**) EV characterization via TEM, protein array and NTA was performed in accordance with the MISEV guidelines. (**D**) Treatment of PC9 cells with pooled Pre- and Post-exercise L-EV enriched fractions. (**E**) RNA isolation and cDNA preparation performed both for characterization of pooled L-EV enriched fraction miRNA cargo and for L-EV–treated PC9 cell miRNA and gene expression analysis. (**F**) Differentially expressed miRNAs (DEmiRs) and genes (DEGs) were further analysed via a pathway analysis pipeline. PPP: Platelet poor plasma; PFP: Platelet-free plasma. Figure created using BioRender.com

This study employed a two-level mixed experimental design integrating a quasi-experimental human exercise intervention with an in vitro cell culture model (Fig. 1).

At the first level, a quasi-experimental pretest–posttest design was used in which healthy, regularly exercising senior males underwent a single bout of acute exercise. After arrival, the participants were asked to spend 15 min in a seated position quietly to recover their calm state. Venous blood samples were collected immediately before (Pre) and after (Post) exercise to assess exercise-induced changes in circulating plasma-derived L-EVs. Physical measurements were taken, and resting heart rate monitoring was completed. Following these measurements, the participants completed an acute exercise test with ECG to determine their cardiorespiratory fitness. The exercise testing was completed at the Institute of Sport Science and Physical Education of the Faculty of Sciences, University of Pécs, Hungary. At this level, the human participants served as their own controls, allowing characterization of exercise-associated alterations in L-EV concentration, size distribution, and molecular cargo.

At the second level, an in vitro experimental design was implemented. L-EV–enriched fractions obtained from pooled Pre- and Post-exercise plasma samples were applied as treatments to PC9 lung adenocarcinoma cells. This experimental component was designed to test whether exercise-conditioned L-EVs differentially modulate tumor-related gene and miRNA expression in recipient cancer cells under controlled conditions. The integration of these two levels enabled mechanistic exploration of exercise-responsive circulating L-EVs while acknowledging that causal inference regarding exercise effects on EV characteristics is limited by the quasi-experimental nature of the human component. Accordingly, the primary mechanistic conclusions of this study derive from the in vitro experimental model, whereas the human exercise intervention provides biological context for L-EV origin.

### Single bout endurance exercise test

The participants were asked to abstain from ingesting food, caffeine, alcohol, or tobacco products within 3 h of testing. Additionally, they were told not to perform strenuous exercise at least 24 h before testing, including a non-exhaustive way to arrive at the laboratory. The participants were urged to consume ample fluid to ensure normal hydration for the day of the tests. To assess the cardiorespiratory status of the participants, a vita maxima spiroergometric test with Bruce ramped treadmill protocol or an incremental cycling test according to the personal preference of participants (Additional file1: Table S2 and S3) was used for volitional exhaustion [41]. Tests were stopped according to the criteria outlined by the American College of Sports Medicine exercise testing guidelines [42]. For exercise intensity control standards and baseline cardiopulmonary matching of groups see Additional file 1 and 2.

### Blood sample collection

Throughout the protocol, we followed the ISTH guidelines for blood sampling and handling for microparticle analysis [43]. Fasting blood collection took place early in the morning (7–7:30 am). The participants were asked to refrain from physical activity 24 h before blood sampling, to drink plenty of water, and drink no caffeine-containing drinks before the day of testing. Venous blood samples were collected in appropriate tubes according to downstream applications: EDTA tubes (routine hematology), native additive-free separator tubes (C-reactive protein, cortisol), and sodium-fluoride containing tubes (glucose). To minimize in vitro thrombocyte-derived exosome release, blood was collected in acidic-citrate-dextrose-B (ACD-B; 1 mL; art. no.: 367756) containing tubes for plasma preparation [43, 44].

### Preparation of serum and plasma

The serum was allowed to clot for 30 min at room temperature before centrifugation. After that, the clotted blood samples were centrifuged for 10 min at 1500 × g, and then the cell-free serum was aliquoted into 1.5 mL microcentrifuge tubes and stored at −80 °C until further use for auxiliary analyses. Blood samples collected in ACD-B tubes were processed within 30 min after sampling to obtain plasma. For platelet-free plasma (PFP) isolation, sequential centrifugation was applied following the ISTH protocol [43] (see Additional file 1 for further detail). The collected PFP samples were aliquoted and stored at −80 °C until further use.

### Isolation and enrichment of L-EVs

Platelet-free plasma (PFP) samples were used for the isolation and enrichment of L-EVs. L-EVs were isolated both from individual PFP samples and from pooled PFP samples using the same standardized protocol. For pooled preparations, 2 ml of PFP from each participant (*n* = 9) were combined to obtain a final volume of 18 ml, which was used for downstream applications in this study (nanoparticle tracking analysis [NTA], EV Protein Array, PCR-based analyses, and cell culture treatments). In parallel, L-EVs were isolated from individual PFP samples to enable participant-level EV characterization, including NTA-based size distribution and particle concentration analyses. PFP sample pools and individual PFP samples were centrifuged at 2000 × g for 20 min to remove any remaining cell debris. The samples were subsequently diluted with 1× phosphate-buffered saline (PBS without Ca and Mg) at a ratio of 1:1 and then filtered through an 800 nm filter without applying pressure. The L-EVs were then pelleted at 20,500 × g for 60 min. The L-EV–free supernatants were carefully aspirated. The pellets were resuspended in 1× PBS and then centrifuged at 20,500 × g for 40 min at room temperature to increase the purity of the isolate. After washing, the supernatant was gently aspirated and discarded. The remaining pellets were stored at − 80 °C until further use.

### Nanoparticle tracking analysis (NTA)

The particle size distribution and concentration were assessed with an NS300 instrument (Malvern, UK) equipped with a blue laser (488 nm) and sCMOS camera. Initially, all the samples were diluted to a final volume of 1 ml in 0.1 μm filtered 1x PBS (pre-checked for the absence of particles). The sample was further diluted to an optimal concentration for measurement (10–50 particles/frame). The settings were set according to the manufacturer’s software manual (NanoSight NS300 User Manual, MAN0541-01-EN-00, 2017). The full acquisition settings are available in Additional file 1. For NTA measurements, particle concentration is reported as mean ± SD, whereas particle size is reported as mode ± SD, as the mode more accurately reflects the peak of the particle size distribution more accurately than the arithmetic mean.

### Protein quantification

Protein quantification of the plasma-derived the L-EV preparation was carried out using Pierce™ BCA Protein Assay kit (Thermo Fisher Scientific) according to the manufacturer’s protocol. Briefly, 25 µl of each standard and sample was pipetted into a microplate in triplicate, and then 200 µl of working reagent was added to each well using a multichannel pipet. The plate was mixed on a shaker for 30 s and then incubated at 37 °C for 30 min. The microplate was then cooled to room temperature, and the absorbance was measured at 562 nm on an EnSight^®^ Multimode Plate Reader (PerkinElmer).

### EV detection with Exo-check

A membrane-based antibody array (Exo-Check™ Exosome Antibody Array, System Biosciences, Palo Alto, CA) was used to detect the presence or absence of 8 known EV markers (FLOT-1, ICAM, ALIX, CD81, CD63, EpCAM, ANXA5 and TSG101) following the manufacturer’s instructions. Briefly, based on the concentrations measured with BCA, 50 µg of L-EV protein was incubated overnight in the array. After washing, detection buffer was added to the membrane. Images of the arrays were captured via a LAS 4000 luminescent analyser (GE Healthcare Bio-Sciences AB Uppsala, Sweden).

### Transmission electron microscopy

Briefly, 2–2.5 µl enriched L-EV suspension was dropped onto a pioloform (1.5%) film-coated single-hole copper grid and allowed to dry horizontally overnight. For enhanced contrast, 2–3 µl of uranyl acetate was added to the films for 15 min before they were washed with distilled water. The grids were then treated with 2–3 µl of lead citrate (Leica Ultrastain 2) for 2–3 min before being washed with distilled water and left to dry at room temperature overnight. Finally, the grids were visualized via a JEOL JEM 1400 FLASH transmission electron microscope.

### Cell culture

EGFR-mutant PC-9 human lung adenocarcinoma cell line (Sigma-Aldrich, St. Louis, Missouri, USA) was cultured in RPMI 1640 (Corning, NY, USA) containing 10% FBS, 1% L-glutamine and 2% penicillin/streptomycin under standard conditions (37 °C, 5% CO₂, pH 7.2–7.4). All experiments were performed with mycoplasma-free cells.

After PC-9 cells were grown in 100% complete RPMI, the cells were adapted to Exosome depleted FBS (System Biosciences, ref. EXO-FBS-50 A-1 lot. 082715) containing medium with sequential decreasing concentration of 10% FBS containing RPMI (Additional file 1: Table S4).

### Cell treatment

PC-9 cells were seeded at a density of 35,000 cells per well in a 24-well plate under three experimental conditions: untreated control (CTRL), Pre-L-EV-treatment, and Post-L-EV-treatment (Additional file 1: Fig. S3). After 24 h, cells were treated with 1 mL of EDRPMI containing 10⁹ nanoparticles (NPs). Following a 24-hour incubation, the cells were harvested by adding 300 µl of trypsin and incubating for 7 min at 37 °C. Trypsinization was stopped by adding complete culture medium, and the cells were collected by centrifugation at 1000 rpm for 5 min at 23 °C. The pellet was washed with ice cold 1x PBS, centrifuged again, and the supernatant was discarded. A total of 150,000 cells were collected per treatment condition and stored as pellets at −80 °C until further use.

### Cell viability and metabolic activity assays

Functional assays were performed under conditions identical to those used for transcriptomic analyses. PC9 cells were seeded at a density of 35,000 cells per well in 24-well culture plates and allowed to attach overnight at 37 °C in a humidified atmosphere containing 5% CO₂. The following day, cells were treated with 10⁹ enriched L-EVs suspended in EDRPMI medium. Three experimental conditions were established: untreated control (CTRL), Pre-L-EV, and Post-L-EV treatment. Cells were incubated for 24 h following treatment.

After incubation, cells were collected and counted using the trypan blue exclusion method (Fig. S4). For ATP-based metabolic activity assessment, equal volumes (100 µL) of cell suspension from each condition were transferred in triplicate into a white 96-well microplate. Background control wells containing medium only were included. The plate was equilibrated to room temperature, followed by the addition of 100 µL CellTiter-Glo^®^ 3D reagent (Promega) to each well. Plates were shaken for 5 min to induce cell lysis and incubated for 25 min at room temperature prior to luminescence measurement using an EnSight^®^ Multimode Plate Reader (PerkinElmer).

For complementary assessment of metabolic activity, equal volumes of cell suspensions (100 µL) were seeded into 96-well plates in triplicate for each condition. Cell viability was evaluated using the CyQUANT™ XTT Cell Viability Assay (Thermo Fisher Scientific) according to the manufacturer’s instructions. Absorbance was measured at 450 nm (signal) and 660 nm (background), and specific absorbance was calculated as:

Specific Absorbance = (Abs₄₅₀ Test − Abs₄₅₀ Blank) − Abs₆₆₀ Test.

### RNA isolation and cDNA synthesis

Total RNA, including small RNA species, was isolated from pooled Pre- and Post-exercise plasma-derived L-EV enriched fractions using the Total Exosome RNA & Protein Isolation Kit (Invitrogen) according to the manufacturer’s protocol. This RNA preparation was used for the analysis of L-EV miRNA cargo.

Total RNA was isolated from treated and control PC9 cells with mirVana™ miRNA Isolation Kit (Invitrogen) according to the manufacturer’s protocol. After the lysis step, 0.1 pmol/µl cel-miR-39 (Phos-UCACCGGGUGUAAAUCAGCUUG) spike-in miRNA with a phosphate modification at the 5’ end was added to each lysate for quality control purposes (Additional file 1: Fig. S5). The concentration of the total RNA isolates was measured using spectrophotometer (Implen NanoPhotometer^®^ N60/N50, Germany).

For miRNA expression quantification cDNA template was prepared from 10 ng total RNA using the TaqMan™ Advanced miRNA cDNA Synthesis Kit (Applied Biosystems) according to the manufacturer’s protocol.

For gene expression quantification in PC9 cells, total RNA concentrations were normalized to the lowest amount. Two micrograms of total RNA was used to generate cDNA using High-Capacity cDNA Reverse Transcription Kit (ThermoFisher Scientific, Waltham, Massachusetts, USA). The cDNA products were stored at −20 °C until qRT-PCR analysis.

### Quantitative (q)RT-PCR

miRNA expression profiling was performed using the TaqMan^®^ Advanced miRNA Human A cards (Applied Biosystems), containing 384 miRNA assays. Pre-amplified cDNA was diluted and loaded with TaqMan^®^ Fast Advanced Master Mix according to the manufacturer’s protocol. The reactions were run on a QuantStudio™ 12k Flex Real-Time PCR System and global mean normalization was applied [45]. Gene expression was assessed using the TaqMan^®^ Array Human Molecular Mechanisms of Cancer panel (Applied Biosystems), which comprises 92 cancer-associated genes and 4 candidate reference genes measured with the QuantStudio™ 12k Flex Real-Time PCR System. GAPDH was selected for normalization, and relative quantification was performed using the comparative C_T_ (ΔΔC_T_) method. The results were analysed via Expression Suite Software v1.3. Gene and miRNA expression profiling was conducted as an exploratory analysis based on a single, standardized treatment condition. Differential expressions were therefore evaluated using fold-change thresholds (≥ 1.5 or ≤ 0.667) to identify biologically meaningful shifts, rather than to support inferential statistical testing. The results were interpreted at the pathway and network level to capture coordinated regulatory effects characteristic of EV-mediated signaling.

### Bioinformatic analysis of miRNA targets, genes and associated pathways

To explore the functional relevance of differentially expressed miRNAs (DEmiRs), target gene prediction was performed using MiRTarBase 2022, a database of experimentally validated miRNA–mRNA interactions. The identified targets were subjected to Kyoto Encyclopedia of Genes and Genomes (KEGG) pathway enrichment using DIANA-miRPath v4.0 [46], with statistical significance set at *p* < 0.05 and FDR correction applied. To further evaluate the relationships among DEmiRs, differentially expressed genes (DEGs) and their predicted cellular effects, Ingenuity Pathway Analysis (IPA, QIAGEN) was used. Separate core analyses were performed for each treatment condition (Pre and Post), incorporating both observed and high confidence predicted interactions. IPA was employed to visualize DEmiR–DEG regulatory networks, assess upstream regulators, and infer activation or inhibition of cancer-relevant cellular functions.

### Statistical analysis

Statistical analyses and data visualization were conducted using SPSS v25 and GraphPad Prism v10. The normality of the data distribution was assessed using the Shapiro–Wilk test. For comparisons between two independent groups, independent samples t-tests were used. Paired t-tests were applied for within-subject comparisons of normally distributed repeated measures. For comparisons involving more than two groups, one-way analysis of variance (ANOVA) was performed, followed by Tukey’s post hoc test for multiple comparisons when significant main effects were detected. Effect sizes for ANOVA were reported using omega squared (ω²). In cases where assumptions of normality were not met, non-parametric alternatives were considered as appropriate. Pearson’s correlation coefficient was used to evaluate linear associations between continuous variables. Effect sizes for pairwise comparisons were reported using Cohen’s d. Statistical significance was defined as *p* ≤ 0.05. The data are presented as mean ± standard deviation (SD) unless otherwise stated.

## Results

### Quasi-experimental human study

#### Anthropometric and physiological characteristics of the participants

The study involved 9 senior endurance-trained participants who met the inclusion criteria. The participant characteristics are provided in Table 1. All participants were non-smokers, in good overall health, and all their physiological parameters were within the age-matched normal range. The relative VO_2_ max values were above the age-matched reference range (see Additional file 2 for further detail). To assess baseline comparability between exercise modalities, cardiopulmonary and physiological parameters were compared between treadmill and cycle ergometer groups using appropriate statistical tests based on data distribution. No significant differences were observed for VO₂max, percentage of predicted VO₂peak, ventilatory efficiency (VE/VCO₂ slope), heart rate indices, or blood pressure at *p* ≤ 0.05, supporting adequate baseline cardiopulmonary matching and justifying pooling of samples for downstream extracellular vesicle analyses (see Additional file 2 for further detail).

**Table 1:** Participants’ characteristics

Characteristics	Mean ± SD
Gender	Male
Age (years)	61 ± 2
Body Fat Percentage (%)	21.29 ± 6.84
VO_2_ max (ml/kg/min)	33.97 ± 7.22
Glucose (mmol/L)	5.69 ± 0.48
Systolic BP (Hgmm)	130.33 ± 15.72
Diastolic BP (Hgmm)	86.89 ± 8.72

VO_2_ max, maximal oxygen uptake (cardiorespiratory fitness); BP (Blood Pressure)

**Table 2:** Lipoproteins and blood cell counts of participants before and after exercise

	HDL (mmol/L)	LDL (mmo/L)	Platelets (G/L)	Erythrocytes (T/L)	Leukocytes (G/L)	Neutrophils (G/L)	Monocytes (G/L)	Lymphocytes (G/L)
Pre-Exercise	1.58 ± 0.48	3.46 ± 1.25	241.89 ± 55.90	5.26 ± 0.35	6.20 ± 1.23	3.37 ± 0.74	0.53 ± 0.15	2.09 ± 0.48
Post-Exercise			277.44 ± 61.91	5.42 ± 0.27	8.64 ± 1.62	4.60 ± 1.01	0.67 ± 0.20	3.16 ± 0.85
Normal values	>0.9	<3.4	140 - 440	4.5 - 6.0	4.0 - 10	1.78 - 5.38	0.3 - 0.82	1.32 - 3.57

#### EV characteristics and exercise effects

**Fig. 2:**
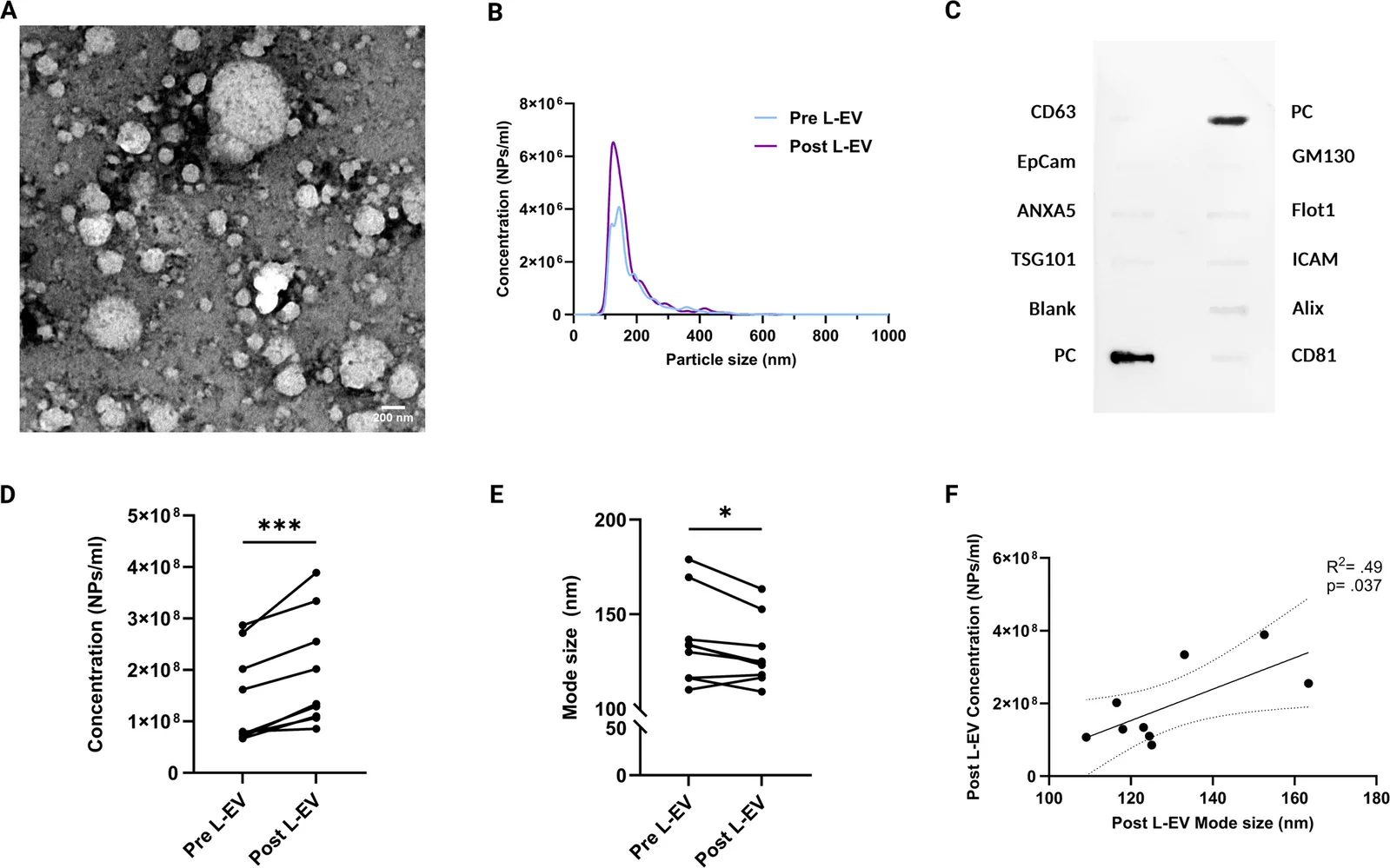
(**A**) TEM image of representative plasma derived L-EV enriched fraction. (**B**) Representative size distribution profile of the EVs in the sample pool of Pre-, and Post-exercise samples. (**C**) Antibody membrane array: Exosome antibody arrays incubated with L-EV enriched fraction lysates confirmed the presence and absence of proteins specific to exosomes (CD63; EpCam: epithelial cell adhesion molecule; ANXA5: Annexin A5; TSG101: tumor susceptibility gene 101; Flot1: flotillin-1; ICAM: intercellular adhesion molecule 1; ALIX: ALG-2-interacting protein X; CD81), and a cellular contamination marker (GM130: cis-Golgi matrix protein) (PC: positive control for HRP detection, Blank: background control). Individual (*n* = 9) nanoparticle (NP) concentrations (**D**) and sizes (**E**) of Pre-, and Post-exercise L-EV enriched samples were measured. Each line represents a paired sample from the same subject (*p < 0.05, ***p < 0.001). (**F**) Linear regression analysis of post-exercise L-EV concentration and size

EV fractions were isolated from platelet-free plasma (PFP) as described in the Materials and Methods. The isolation and enrichment protocol for L-EVs was validated via multiple approaches. Transmission electron microscopy (TEM) confirmed the expected size and morphology of the vesicles. A representative image (Fig. 2A) shows typical L-EVs with diameters exceeding 200 nm. Nanoparticle tracking analysis (NTA) was also performed on pooled pre- and post-exercise samples (Pre-L-EV and Post-L-EV) to generate representative size distribution profiles (Fig. 2B). The pooled pre-exercise sample had a particle concentration of 3.53 × 10⁸ ± 1.89 × 10⁷ particles/mL with a mode size of 143.5 ± 81.5 nm, whereas the post-exercise sample had a concentration of 5.01 × 10⁸ ± 3.74 × 10⁷ particles/mL and a mode size of 125.2 ± 74.7 nm. EV protein profiling was conducted using exosome antibody array membranes containing eight canonical EV markers (CD63, CD81, EpCam, ANXA5, TSG101, FLOT-1, ICAM, and ALIX) (Fig. 2C). It revealed weak signals for canonical exosomal markers and GM130 that were clearly distinguishable from the positive control but slightly above background levels.

Individual quantification of participant samples via NTA further supported these results (see Additional file 2 for further detail). The plasma nanoparticle concentration increased significantly following an acute bout of endurance exercise, with an average increase of 5.05 × 10⁷ ± 9.99 × 10⁶ particles/mL (t(8) = 5.1, *p* < 0.001) and a large and robust effect size (Cohen’s d = 1.685) (Fig. 2D). Although only the treadmill group showed a statistically significant increase in L-EV concentration (t(4) = 10.87, *p* < 0.001, d = 4.861) no significant differences were detected between exercise modalities in the values of pre- exercise (t(7) = 1.004, *p* = 0.349, d = 0.673) and post- exercise (t(7) = 0.741, *p* = 0.483, d = 0.497) values (Additional file 1: Fig. S6). The particle size dynamics were also assessed. The NTA results confirmed that the purified particles resembled L-EVs with particle sizes of 136.1 ± 23.6 nm pre-exercise and 129.5 ± 17.7 nm post-exercise [11]. A paired samples t-test revealed that this decrease was statistically significant (t(8) = 2.634, *p* = 0.030), with a mean difference of 6.67 nm and a large effect size (Cohen’s d = 0.878) (Fig. 2E). No significant differences were detected between exercise modalities regarding pre- and post-exercise particle size (*p* > 0.05, Additional file 2). TEM predominantly visualized vesicles with diameters exceeding 200 nm, whereas NTA analysis identified a particle size peak near 130 nm. TEM visualizes dehydrated, surface-adsorbed vesicles, which accentuates larger intact structures, whereas NTA measures hydrodynamic diameter in suspension and often underestimates size in heterogeneous populations ─ particularly when larger vesicles partially fragment during processing. A moderate positive correlation was observed between particle mode size and concentration in the case of Post-L-EV (F(1, 7) = 6.6, *p* = 0.037, R² = 0.49; Fig. 2F).

All evaluated blood parameters that could have interfered with changes in EV concentration were within the normal physiological range at rest and did not change during exercise (Table 2), confirming the participants’ health status. Finally, while the presence of co-isolated lipoproteins cannot be ruled out [29], the significant negative correlation between Pre-L-EV count and HDL concentration (r(7) = − 0.782, *p* = 0.012; see Additional file 2 for further detail).

#### L-EV enriched fractions show distinct miRNA profiles at baseline and after a single bout of exercise

qRT-PCR with advanced TaqMan chemistry was used to determine the miRNA profile of enriched L-EVs from pooled plasma samples and treated cell samples. miRNAs were considered differentially expressed if the FC was ≥ 1.5 or ≤ 0.667.

**Fig. 3:**
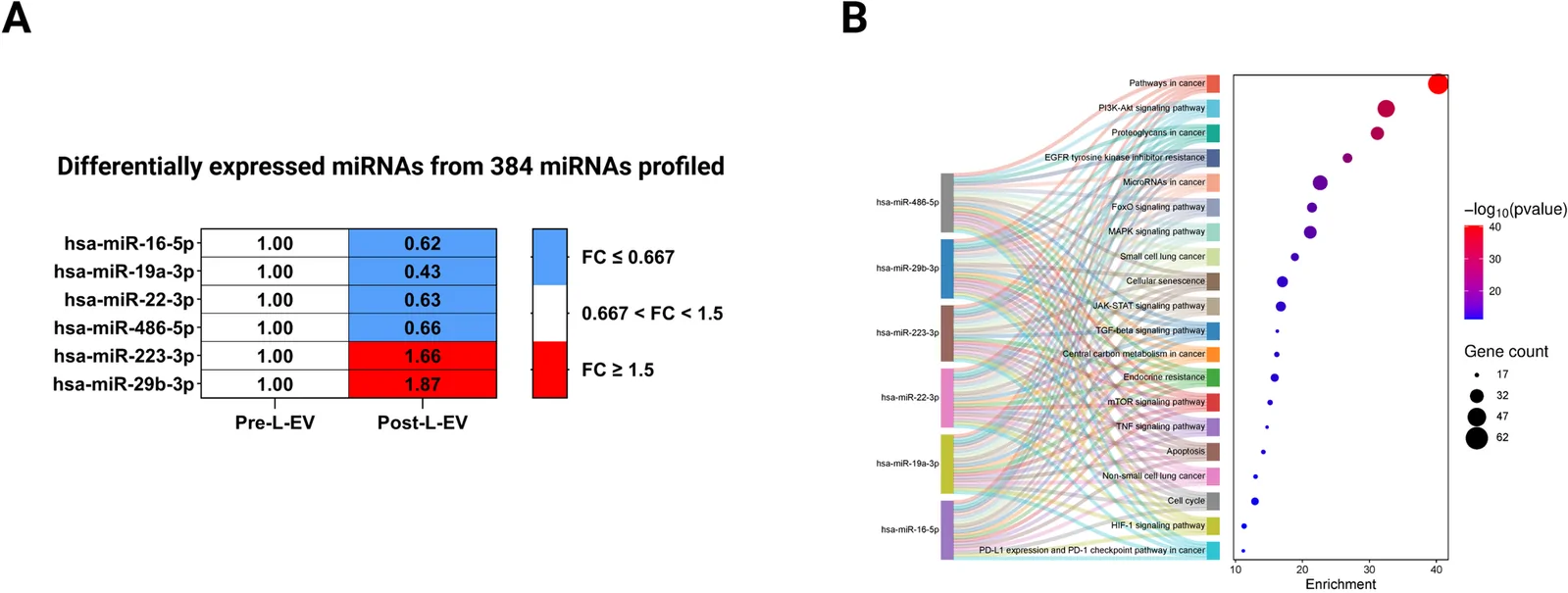
Exercise-induced changes in the large EV enriched fraction’s miRNA cargo and enrichment of cancer-related pathways. (**A**) Heatmap showing the differentially expressed miRNAs in plasma-derived large EV (L-EVs) enriched fractions before (Pre-L-EV) and immediately after (Post-L-EV) acute exercise, identified using the TaqMan™ Advanced miRNA Human A Card (384 assays). Only miRNAs passing the differential expression threshold (FC ≥ 1.5 or ≤ 0.667) are displayed. The values represent the fold change relative to Pre-L-EV (Pre-L-EV was set to 1.00). Blue indicates downregulation (FC ≤ 0.667) and red indicates upregulation (FC ≥ 1.5) in Post-L-EV. (**B**) Integrated pathway enrichment analysis of the predicted mRNA targets of the six differentially expressed miRNAs. Left: Sankey diagram linking each miRNA to enriched KEGG pathways (top 20 ranked by significance, cancer-relevant). Right: Bubble plot displaying the top 20 enriched pathways on the vertical axis, and the horizontal axis represents the enrichment. The colour of the bubble indicates enrichment significance (–log10 (p value)). The smaller the p-value is, the closer the colour is to red. The number of genes targeted by the differentially expressed miRNAs included in each pathway is expressed by the size of the bubbles

We first profiled miRNAs in L-EV enriched fractions isolated from plasma before (Pre-L-EV) and immediately after (Post-L-EV) an acute bout of exercise (Fig. 3A). Among the six miRNAs detected, four (hsa-miR-16-5p, hsa-miR-19a-3p, hsa-miR-22-3p, and hsa-miR-486-5p) were significantly downregulated post-exercise (FC ≤ 0.667), whereas two (hsa-miR-223-3p, and hsa-miR-29b-3p) were upregulated (FC ≥ 1.5). To explore the functional implications of these shifts, we performed target prediction for the six differentially expressed miRNAs and extracted the top 20 KEGG pathways enriched among their predicted mRNA targets, focusing on cancer-relevant processes (Fig. 3B and Additional file 2). Notably, the “Pathways in cancer” category exhibited the strongest enrichment (enrichment score ≈ 40, 62 target genes), followed by “PI3K-Akt signaling,” “Proteoglycans in cancer,” “EGFR tyrosine kinase inhibitor resistance,” and “MicroRNAs in cancer.” Several lung cancer-specific pathways (e.g., “Non-small cell lung cancer,” “Small cell lung cancer”) and key oncogenic signaling axes (MAPK, Ras, p53, TNF, HIF-1) also ranked among the most significant (–log₁₀ p values ≥ 10).

### In vitro experimental study

#### Exercise-conditioned L-EVs reduce metabolic activity in PC9 cells

To assess whether transcriptional changes were accompanied by functional effects, metabolic activity of PC9 cells was evaluated following treatment with Pre- and Post-exercise enriched L-EVs.

**Fig. 4:**
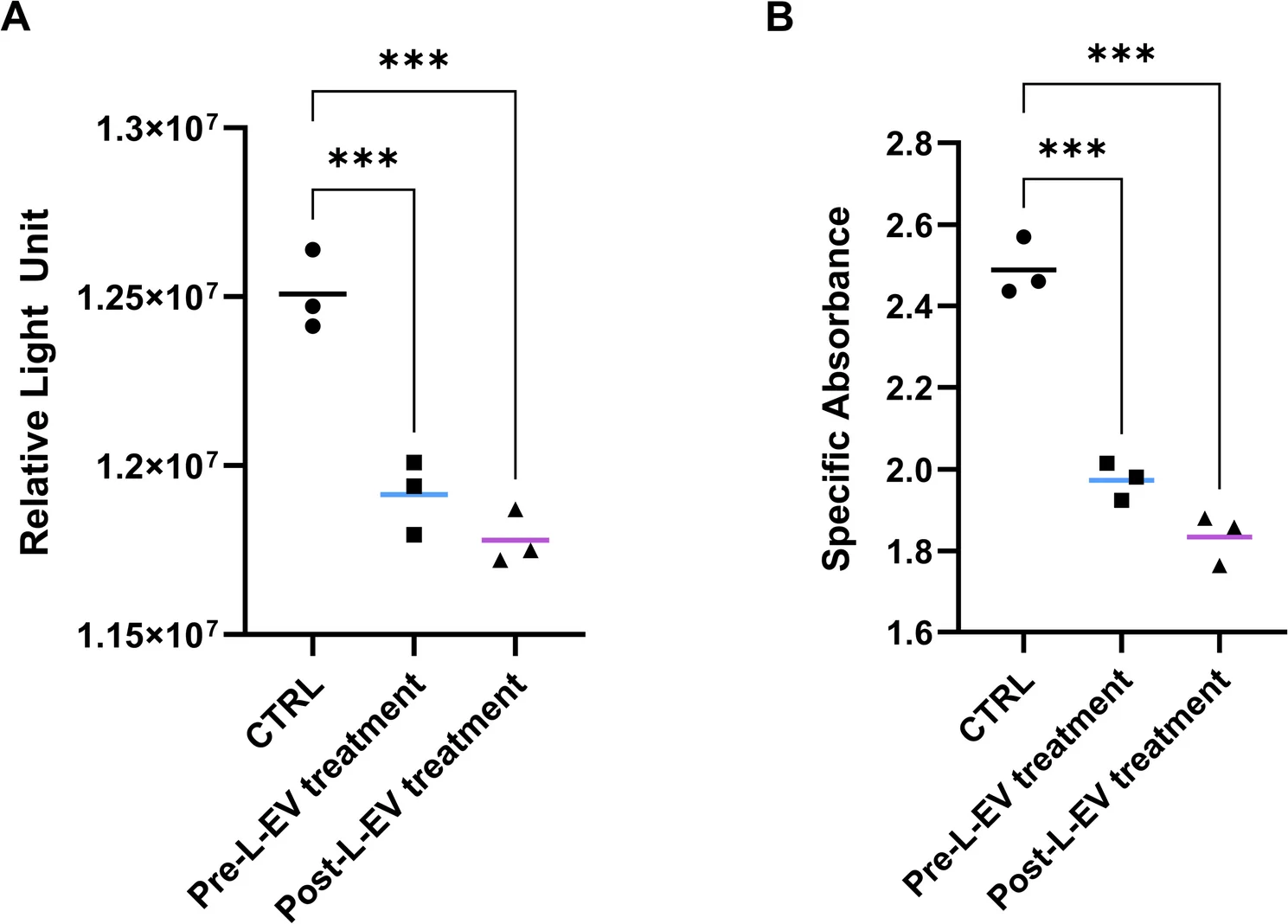
Metabolic activity of PC9 cells following 24 h treatment with Pre- and Post-exercise L-EVs. (**A**) ATP-based luminescence assay (CellTiter-Glo^®^) showing relative metabolic activity across conditions. (**B**) XTT assay measuring mitochondrial metabolic activity via absorbance. Data are presented as mean ± SD (*n* = 3 per condition). Statistical significance was assessed using one-way ANOVA followed by Tukey’s post hoc test (***p < 0.001 vs. CTRL)

ATP-based luminescence measurements revealed a significant reduction in metabolic activity in both treatment groups compared to untreated controls (CTRL: 1.25 × 10⁷ ± 1.17 × 10⁵; Pre: 1.19 × 10⁷ ± 1.09 × 10⁵; Post: 1.18 × 10⁷ ± 7.95 × 10⁴). One-way ANOVA indicated a significant overall effect (F(2,6) = 42.51, *p* < 0.001, ω² = 0.902). Post hoc analysis confirmed significant differences between CTRL and both Pre- and Post-L-EV treatments (*p* < 0.001), while no significant difference was observed between Pre- and Post-L-EV groups (Fig. 4A).

Consistently, XTT-based metabolic activity measurements showed a significant decrease in treated cells (CTRL: 2.49 ± 0.07; Pre: 1.97 ± 0.05; Post: 1.83 ± 0.06). One-way ANOVA confirmed a significant effect across conditions (F(2,6) = 98.04, *p* < 0.001, ω² = 0.956), with post hoc comparisons indicating significant reductions in both treatment groups compared to control (*p* < 0.001). The difference between Pre- and Post-L-EV treatments did not reach statistical significance (Fig. 4B).

#### Differential miRNA and gene remodelling of PC9 cells by pre- and post-exercise L-EV enriched fractions

**Fig. 5:**
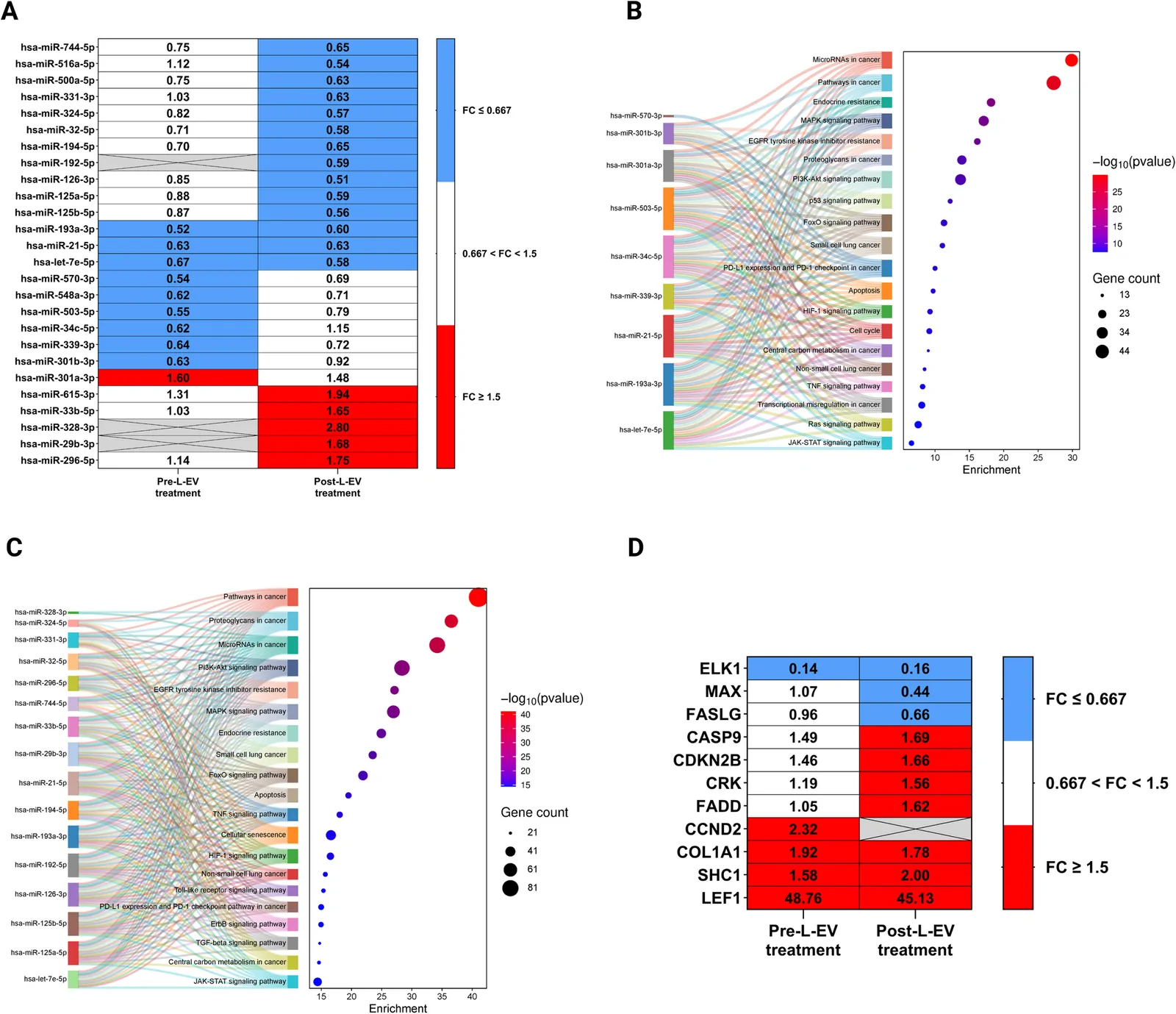
Exercise-conditioned enriched L-EVs reprogram PC9 cell miRNA profiles and modulate cancer-related pathways and gene expression. (**A**) Heatmap showing the fold changes (FCs) of 25 differentially expressed miRNAs (DEmiRs) in PC9 cells treated with L-EV enriched fractions collected before (Pre-L-EV) and after (Post-L-EV) acute exercise relative to those in untreated controls. Blue shading indicates downregulation (FC ≤ 0.667), and red shading indicates upregulation (FC ≥ 1.5). (**B**) Bubble plot of the top 20 KEGG pathways enriched among the predicted targets of Pre-L-EV–responsive miRNAs. The x-axis represents the enrichment score; the bubble size corresponds to the number of target genes, and the bubble colour indicates the –log₁₀ (p-value). (**C**) Bubble plot of the top 20 KEGG pathways enriched among the predicted targets of Post-L-EV–responsive miRNAs. The plot conventions are the same as those in (B). (**D**) Differential expression of cancer-related genes in PC9 cells following treatment with enriched Pre- and Post-L-EVs. The heatmap displays genes that met the predefined differential expression criteria following enriched L-EV treatment. These genes were selected from a targeted panel of 92 cancer-associated genes encompassing multiple oncogenic signaling pathways

To investigate how plasma-derived L-EV enriched fractions influence gene regulation in lung cancer, we treated PC9 cells with L-EV enriched fractions isolated from healthy individuals before (Pre-L-EVs) and after (Post-L-EVs) an acute bout of physical exercise. Following a 24 h incubation, total RNA was extracted from PC9 cells, and miRNA and gene expression profiles were analysed via RT-qPCR. This revealed multiple DEmiRs and DEGs under both treatment conditions, with the Post-L-EV treatment inducing a more extensive and distinct transcriptional response compared to the untreated controls (Fig. 5A).

Under Pre-L-EV conditions, nine miRNAs were significantly downregulated (hsa-miR-193a-3p, −21-5p, let-7e-5p, −570-3p, −548a-3p, −503-5p, −34c-5p, −339-3p, and − 301b-3p; FC ≤ 0.667) and one miRNA was upregulated (hsa-miR-301a-3p; FC ≥ 1.5). In contrast, Post-L-EV treatment expanded this regulatory footprint to fourteen downregulated (hsa-miR-744-5p, −516a-5p, −500a-5p, −331-3p, −324-5p, −32-5p, −194-5p, −192-5p, −126-3p, −125a-5p, −125b-5p, −193a-3p, −21-5p, and let-7e-5p) and six upregulated miRNAs (hsa-miR-615-3p, −33b-5p, −328-3p, −29b-3p, and − 296-5p). Only three miRNAs overlapped and changed in the same direction between Pre- and Post-conditions, highlighting a small, shared core effect and a pronounced increase in L-EV-responsive miRNAs after exercise.

To explore the functional impact of these miRNA shifts, we performed KEGG pathway enrichment on their predicted targets, focusing on cancer- and lung cancer–relevant processes (see Additional file 2 for further detail). Pre-L-EV targets (Fig. 5B) were enriched in “MicroRNAs in cancer”, “Pathways in cancer”, “PI3K-Akt signaling”, “Proteoglycans in cancer”, “EGFR tyrosine kinase inhibitor resistance”, “p53 signaling” and “Cell cycle” (–log₁₀ p up to ~ 30; gene counts 16–55), indicating the modulation of major oncogenic and proliferative networks. Post-L-EV targets (Fig. 5C) retained these canonical pathways but also showed strong enrichment for “Apoptosis”, “Central carbon metabolism in cancer”, “PD-L1 expression and PD-1 checkpoint” and “Non-small cell lung cancer” (–log₁₀ p up to ~ 40; gene counts 22–102).

Among the 92 evaluated cancer-associated genes, *ELK1*, *MAX* and *FASLG* were significantly downregulated after Post-L-EV exposure, with *ELK1* remaining consistently suppressed across both conditions (Fig. 5D). In contrast, several genes including *CASP9*, *CDKN2B*, *CRK*, and *FADD* were upregulated only under postconditioning conditions, whereas *CCND2*, *COL1A1*, *SHC1* and *LEF1* presented increased expression across both treatment conditions relative to the control. For the full list of evaluated lung-adenocarcinoma relevant genes see Table S5.

To further interpret the molecular changes the DEmiRs and DEGs were uploaded into IPA to assess overrepresented biological pathways. Under the Pre-L-EV treatment conditions, we observed enrichment in canonical pathways associated with oncogenic and proliferative signaling, including “Thyroid Cancer”, “IL-6, and Glioblastoma Multiforme signaling”, as well as “Erythropoietin and activin signaling”. The disease profile associated with Pre-L-EV treatment included organismal injury, reproductive system disorders, cancer, neurological diseases, and psychological disorders. The enriched cellular functions included movement, development, proliferation, cell cycle regulation, and cell death and survival, with notable associations with hematological system development, hematopoiesis, lymphoid tissue structure, tissue development, and cardiovascular function.

Conversely, Post-L-EV treatment resulted in a shift toward pathways that favour anti-tumor and apoptotic responses. Key pathways enriched in Post-L-EV included “Molecular mechanisms of cancer”, “MYC-mediated apoptosis signaling”, “Tumoricidal functions of hepatic natural killer cells”, “p38 MAPK, and FasL/CD95L signaling”. Although cancer and organismal injury were still present in the disease profile, additional associations with reproductive system diseases, connective tissue disorders, and inflammatory conditions emerged. Cellular functions such as development, growth, movement, and cell cycle progression remained prominent, whereas associations with skeletal and muscular system development were specifically observed in the Post-L-EV condition.

#### Regulatory interactions and predicted functional outcomes in PC9 cells following L-EV enriched fraction treatment

**Fig. 6:**
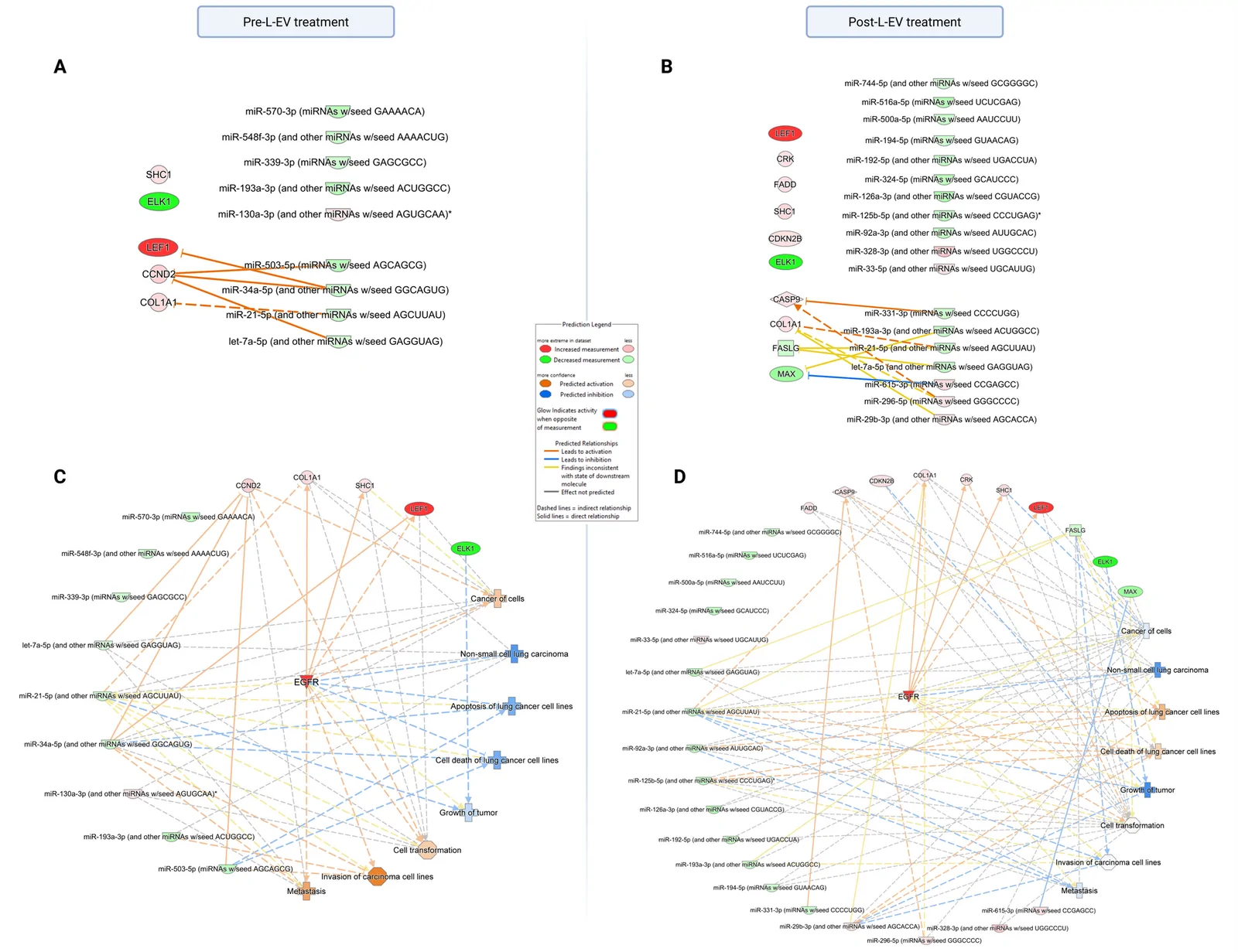
Predicted DEmiR–DEG interactions following pre- (**A**) and post- exercise (**B**) L-EV treatments. Predicted effects of DEGs and DEmiRs on cancer-related functions in PC9 cells following enriched pre- (**C**) and post-exercise (**D**) L-EV treatments

Using IPA, we assessed the potential regulatory relationships between DEmiRs and DEGs in PC9 cells treated with plasma-derived L-EV enriched fractions (Fig. 6A-B). Under both treatment conditions, only a small subset of DEmiRs were predicted to interact with DEGs via experimentally validated or highly predicted interactions, conversely, the majority of DEGs lacked regulatory connections to the altered miRNAs. This disconnect was especially apparent after Post-L-EV treatment, where 6 out of the 10 DEGs did not show any association with the significantly modulated miRNAs (Fig. 6B).

After exploring the limited number of links between DEmiRs and DEGs, we used IPA to predict how the combined expression changes might affect key cellular functions associated with cancer. To mimic PC9 characteristics, EGFR was set as being upregulated for all analyses. As shown in Fig. 6C-D, the functional predictions markedly differed between the treatments. Under Pre-L-EV conditions (Fig. 6C), cancer-promoting processes such as “Cancer of cells”, “Cell transformation”, “Invasion of carcinoma cell lines” and “Metastasis” were predicted to be activated, whereas “Non-small cell lung carcinoma”, “Apoptosis of lung cancer cell lines”, “Cell death of lung cancer cell lines” and “Growth of tumor” were predicted to be inhibited. In contrast, exposure to enriched Post-L-EVs ─ derived from acutely exercised plasma ─ was associated with a reversal of several of these predictions (Fig. 6D). Specifically, functions related to tumor suppression, including “Apoptosis of lung cancer cell lines” and “Cell death of lung cancer cell lines” were predicted to be activated, whereas cancer-promoting processes such as “Cancer of cells”, “Non-small cell lung carcinoma”, “Invasion of carcinoma cell lines”, “Metastasis” and “Growth of tumor” were predicted to be inhibited.

## Discussion

The results of our study revealed that L-EV enriched fractions isolated from the plasma of healthy, regularly exercising senior individuals before and after an acute exercise stimulus differentially affect and alter miRNA and gene expression in PC9 lung adenocarcinoma cells. Notably, treatment with enriched Post-L-EVs resulted in gene expression changes consistent with increased apoptotic signaling and inhibition of cell cycle progression ─ which aligns with the known anti-carcinogenic effect of regular physical activity. Consistent with these transcriptional changes, functional assays demonstrated a significant reduction in metabolic activity in PC9 cells following treatment with both enriched Pre- and Post-L-EVs, supporting the biological relevance of the observed molecular remodeling.

In addition to altering vesicle bioactivity, acute exercise significantly increased the concentration of circulating plasma-derived L-EVs and modestly shifted their size distribution toward smaller mode diameters. This pattern is consistent with an exercise-induced enhancement of vesicle release and/or shedding, likely reflecting an adaptive physiological response to acute systemic stress. The observed reduction in mode size may indicate preferential release of smaller L-EV subpopulations or altered vesicle biogenesis dynamics; however, alternative explanations such as mechanical fragmentation or in-circulation remodelling of the vesicular corona cannot be fully excluded. Importantly, baseline blood parameters remained stable and within normal physiological ranges, supporting the conclusion that the observed increase in circulating L-EVs was not driven by changes in blood cell counts, but instead likely reflects vesicle release from exercise-responsive tissues such as skeletal muscle, vascular endothelium, or immune cells. In biological terms, our results suggest that acute physical activity affects not only the concentration and size distribution of circulating nanoparticles, but also the bioactive cargo of L-EVs, thereby enhancing their potential to influence tumor cell behaviour. Following Pre-L-EV treatment, several proliferation-associated genes (*CCND2*, *COL1A1*, *SHC1*, and *LEF1*) were upregulated, whereas *ELK1* expression was markedly reduced under both treatment conditions. In contrast, Post-L-EV treatment induced further upregulation of pro-apoptotic genes (*FADD*, *CASP9*, and *CDKN2B*), along with the downregulation of *MAX* and *FASLG*, two genes with established roles in oncogenic transcription and immune evasion.

At the systemic level, acute exercise also remodelled the miRNA cargo of circulating enriched L-EVs. Although only a limited number of miRNAs were differentially expressed in plasma-derived enriched L-EVs, pathway enrichment analysis revealed a strong convergence of their predicted targets on canonical cancer-related pathways, including PI3K–Akt, MAPK, Ras, p53, and lung cancer–specific signaling networks. This suggests that even subtle, coordinated changes in L-EV-associated miRNAs may exert biologically meaningful effects by targeting central regulatory hubs, thereby providing a mechanistic link between acute exercise and downstream modulation of tumor-related signaling pathways.

The observed reduction in *MAX* may represent a favourable shift in the transcriptional regulatory balance within the MYC/MAX axis. MAX forms heterodimers with MYC to promote the transcription of genes involved in proliferation, metabolic adaptation, and immune evasion [47, 48]. Its suppression may disrupt this interaction, thereby weakening MYC-driven oncogenic signaling and potentially enhancing cellular sensitivity to targeted EGFR therapies ─ which is especially relevant in EGFR-mutant tumor types such as PC9 [49].

Similarly, the downregulation of *FASLG* in the Post condition may indicate a reduction in the tumor’s capacity for immune evasion via the Fas counterattack mechanism [50]. High FasL expression enables cancer cells to induce apoptosis in cytotoxic immune cells, contributing to immune suppression and resistance to immunotherapies [50–52]. Reduced *FASLG* expression may thus restore immune surveillance and support anti-tumor immunity. In parallel, the suppression of *ELK1* was consistent across both conditions and may represent an important tumor-suppressive shift. *ELK1* is known to increase resistance to EGFR-TKIs and promote epithelial-mesenchymal transition and metastasis in NSCLC [53, 54]. Its downregulation in PC9 cells may therefore improve responsiveness to EGFR inhibition and reduce metastatic potential by impairing oncogenic miRNA networks and EMT-promoting gene programs.

These gene expression results are further supported by the miRNA profile observed in our study. Table 3 summarizes the expression patterns and previously reported functional roles of these miRNAs in NSCLC, providing context for their potential involvement in the observed transcriptional changes. Several oncomiRs previously shown to be upregulated in NSCLC tissue ─ including miR-21-5p, miR-301b, and miR-570-3p ─ were significantly downregulated following enriched L-EV treatment [55–58]. Concurrently, a large subset of tumor-suppressor miRNAs such as let-7e-5p, miR-193a-3p, miR-125a/b-5p, and miR-126-5p were also reduced, likely reflecting broader reprogramming of post-transcriptional regulation in recipient PC9 cells. Importantly, the Post-L-EV treatment induced the upregulation of several tumor-suppressor miRNAs (miR-296-5p, miR-328-3p, miR-33b-5p, and miR-29b-3p) that are commonly downregulated in NSCLC and known to restrict proliferation, migration, and epithelial-mesenchymal transition [59–64]. These changes further corroborate the anti-cancer potential of exercise-conditioned EVs and suggest that their bioactive miRNA cargo plays a central role in mediating tumor-suppressive signaling. These results are in line with a growing body of literature suggesting that regular physical activity reduces the risk of various cancers ─ including lung cancer ─ and improves survival outcomes [34, 65–67]. While most previous studies focused on serum- or plasma-derived free-circulating miRNAs or small EVs, our work highlights the bioactivity of plasma-derived L-EVs, a less explored area with mechanistically promising features.

**Table 3:** Overview of expression changes and functional roles of DEmiRs in non-small cell lung cancer (NSCLC)

**miRNA**	**Sample Source**	**Expression in NSCLC**	**Expression in present Study**	**Known Function**	**Likely Role**	**Ref.**
miR-570-3p	Tumor vs Adj. normal tissue	↑ Upregulated	↓ Downregulated	↓ KLF9, promotes NSCLC cell proliferation	OncomiR	[56]
miR-339-3p	NSCLC vs Healthy serum	↑ Upregulated	↓ Downregulated	Not specified	Unclear	[68]
miR-503-5p	LUAD vs Normal	↑ Upregulated	↓ Downregulated	Suppresses proliferation, regulates apoptosis via Bcl-2	Tumor suppressor	[69, 70]
miR-34a-5p	Tumor vs Adj. normal tissue	↓ Downregulated	↓ Downregulated	Inhibits PD-L1, enhances immune therapy	Tumor suppressor	[71, 72]
miR-548f-3p	—	No data	↓ Downregulated	Not specified	Unclear	—
miR-301a	Tumor vs Adj. normal tissue	↑ Upregulated	↑ Upregulated	Promotes proliferation, migration, invasion	OncomiR	[55]
miR-301b	Tumor vs Adj. normal tissue	↑ Upregulated	↓ Downregulated	Promotes proliferation, migration, invasion	OncomiR	[55]
miR-193a-3p	Tumor vs Healthy tissue	↓ Downregulated	↓ Downregulated	Suppresses proliferation, migration, metastasis	Tumor suppressor	[73, 74]
miR-21-5p	Tumor vs Adj. normal tissue	↑ Upregulated	↓ Downregulated	Inhibits PTEN, promotes growth/metastasis	OncomiR	[57, 58, 75]
let-7e-5p	Tumor vs Normal tissue, NSCLC vs Healthy serum	↓ Downregulated	↓ Downregulated	Targets RAS, MYCN, inhibits proliferation	Tumor suppressor	[76]
miR-744-5p	Tumor vs Adj. normal tissue	↓ Downregulated	↓ Downregulated	Promotes malignancy and therapy resistance	OncomiR	[77, 78]
miR-516a-5p	Tumor vs Adj. normal tissue	↓ Downregulated	↓ Downregulated	Suppresses viability and growth	Tumor suppressor	[79, 80]
miR-500a-5p	Tumor vs Adj. normal tissue	↑ Upregulated	↓ Downregulated	Promotes proliferation, migration; inhibition enhances apoptosis	OncomiR	[81]
miR-194-5p	Tumor vs Normal cell, NSCLC vs Healthy serum	↑ Upregulated	↓ Downregulated	Inhibits proliferation and metastasis via FOXA1	Tumor suppressor	[82]
miR-192-5p	NSCLC vs Normal cell	↓ Downregulated	↓ Downregulated	Decrease cell viability and growth, increases apoptotic rate	Tumor suppressor	[83]
miR-324-5p	Tumor vs Adj. normal tissue	↑ Upregulated	↓ Downregulated	Overexpression increases proliferation, invasion	OncomiR	[84]
miR-126-5p	Tumor vs Normal tissue	↓ Downregulated	↓ Downregulated	Low levels linked to metastasis and late TNM stage	Tumor suppressor	[85]
miR-125a-5p	Tumor vs Normal tissue, NSCLC vs Healthy serum	↓ Downregulated	↓ Downregulated	Loss linked to poor differentiation	Tumor suppressor	[76]
miR-125b-5p	Tumor vs Normal tissue	↓ Downregulated	↓ Downregulated	Inhibits proliferation, migration, invasion; induces apoptosis	Tumor suppressor	[86]
miR-32-5p	Tumor vs Normal tissue	↓ Downregulated	↓ Downregulated	Inhibits migration, invasion	Tumor suppressor	[87]
miR-328-3p	Tumor vs Normal tissue	↓ Downregulated	↑ Upregulated	Restricts proliferation, migration; enhances apoptosis	Tumor suppressor	[60]
miR-33b-5p	Tumor vs Normal tissue	↓ Downregulated	↑ Upregulated	Inhibits proliferation, migration, EMT	Tumor suppressor	[61]
miR-331-3p	Tumor vs Normal tissue, Tumor vs Normal cell	↓ Downregulated	↓ Downregulated	Inhibits proliferation, migration, invasion	Tumor suppressor	[88]
miR-615-5p	Tumor vs Normal tissue, Tumor vs Normal cell	↓ Downregulated	↑ Upregulated	Suppresses cell growth, proliferation, metastasis	Tumor suppressor	[89, 90]
miR-296-5p	Tumor vs Normal tissue, Tumor vs Normal cell	↓ Downregulated	↑ Upregulated	Inhibits migration, PI3K/AKT/mTOR; targets PLK1	Tumor suppressor	[59, 64]
miR-29b-3p	Tumor vs Normal tissue, Tumor vs Normal cell	↓ Downregulated	↑ Upregulated	Inhibition promotes proliferation, migration, invasion	Tumor suppressor	[62, 63]

This table summarizes the differential expression of 30 miRNAs from our study in NSCLC based on previously published datasets and results. For each miRNA, we report the direction of regulation in NSCLC tissues, cells or biofluids compared to non-cancer controls, the sample source, its expression trend in our dataset, and known functional roles as tumor suppressors or oncomiRs. Arrows (↑/↓) indicate upregulation or downregulation, respectively. “—” indicates data not available. Functional annotations are based on literature reports describing effects on proliferation, invasion, apoptosis, immune modulation, or therapeutic resistance

Notably, Pre-L-EV treatment induced measurable molecular changes in PC9 cells, suggesting that the basal extracellular vesicle profile of regularly exercising individuals already has anti-tumor potential. This observation is consistent with the long-term endurance training history of our cohort, whose circulating EV landscape likely reflects chronic physiological adaptation rather than an untrained baseline state. In this context, the additional exercise-induced changes observed under post-exercise conditions elicited more pronounced and favorable gene and miRNA expression shifts. Although the number of differentially expressed miRNAs in plasma-derived L-EVs was modest, this pattern aligns with both the known biology of L-EVs and the characteristics of a long-term trained population. Prolonged physiological conditioning may attenuate the magnitude of acute miRNA responses ─ analogous to exercise hormesis ─ resulting in subtler but tightly regulated vesicle-mediated signaling shifts rather than large, transient fluctuations. Together, these considerations support the interpretation that the observed molecular changes reflect regulated, exercise-related signaling rather than technical limitations.

In this exploratory study, DEmiRs and DEGs were identified primarily based on fold-change thresholds to capture biologically relevant shifts in vesicle-mediated signaling, acknowledging that modest but coordinated miRNA alterations can exert substantial regulatory effects in network-based systems even when individual changes do not reach conventional statistical significance. Regulatory linkage analyses between DEmiRs and DEGs revealed that most differentially expressed miRNAs did not directly correspond to measurable changes in gene expression, particularly in the Post-L-EV-treated cells. This dissociation between individual miRNA abundance and corresponding gene expression changes reflects the inherently complex and cooperative nature of miRNA-mediated regulation. miRNAs rarely act in isolation; instead, they function as interconnected regulatory networks in which multiple miRNAs ─ including those not reaching formal differential expression thresholds ─ collectively fine-tune transcriptional programs. Consequently, single-miRNA overexpression or knockdown approaches may fail to capture the integrated biological effects elicited by a multifactorial extracellular vesicle cargo. In the context of exercise-conditioned L-EVs, the observed phenotypic and transcriptional shifts cannot be attributed solely to changes in individual miRNAs, but rather to coordinated regulation involving multiple miRNAs in concert with other bioactive vesicular components such as proteins, lipids, and metabolites. This network-level regulation, supported by pathway-level concordance in Ingenuity analyses, underscores the importance of systems-level interpretation when evaluating the tumor-modulatory effects of exercise-induced extracellular vesicles.

Although previous experimental work has relied exclusively on small EV-enriched preparations in murine cancer models, the biological direction of their findings is consistent with the tumor-modulatory patterns observed in our human L-EV study. Sadovska et al. [36] reported that exercise-induced small EVs reduced primary tumor growth and attenuated metastatic spread in a prostate cancer model, while Mlynska et al. [37] demonstrated that exercise-responsive small EVs suppressed tumor progression and enhanced anti-tumor immunity in a model of triple-negative breast cancer. Neither study performed broad miRNA or transcriptomic profiling, analyzing either no molecular markers (Sadovska) or only a limited panel of T-cell activation and exhaustion genes (*Eomes*,* Ifng*,* Gzmb*,* Pdcd1*,* Il10*) (Mlynska). Despite these methodological constraints, both studies revealed apoptosis-promoting, proliferation-inhibiting, and immune-supportive effects ─ mechanistic themes that parallel the transcriptomic shifts induced by exercise-conditioned L-EV enriched fractions in our PC9 cells. Our data extends these results by providing the first human-derived mechanistic evidence that exercise-conditioned enriched L-EVs can reprogram cancer cell miRNA and gene expression profiles toward tumor-suppressive outcomes, suggesting that vesicle-mediated anti-cancer signaling is a conserved feature of exercise adaptation across EV subtypes and experimental models.

This study has, several limitations, including the exclusive inclusion of male participants. Although this improved cohort homogeneity and matched the male origin of the PC9 cell line, future work studies should include adequately powered female cohorts to explore potential sex-specific exercise-induced L-EV responses. As this work represents a pilot mechanistic study requiring pooled L-EV enriched preparations to meet assay input requirements, the results should be interpreted as exploratory. Importantly, pooling plasma-derived L-EV enriched samples inherently masks inter-individual variability and results in an averaged vesicle profile, thereby limiting the ability to assess biological heterogeneity or individual response patterns, which may be relevant for translational applications. Future studies integrating functional assays with multi-omics profiling in larger, individual-level cohorts will be essential to confirm these results, establish causality, and quantify the magnitude of L-EV-induced phenotypic effects. In addition, protein-level validation of key targets was not performed in the present study; therefore, future investigations incorporating proteomic or targeted protein assays will be important to confirm the translation of transcriptomic changes into functional signaling outcomes. Furthermore, because the results are based on a single lung adenocarcinoma cell line, validation across additional models will be necessary to confirm generalizability. A more comprehensive omics analysis of L-EV cargo and downstream cellular responses may also help to uncover more subtle contributors beyond the DEmiR and DEG profiles reported in the present study. Finally, although our relative quantification approach revealed robust expression changes, absolute quantification techniques may pinpoint more subtle differences.

## Conclusions

In summary, exercise-conditioned plasma-derived enriched L-EVs elicited coordinated changes in tumor-relevant gene and miRNA expression in lung adenocarcinoma cells. These results provide preliminary mechanistic insight into how regular physical activity may contribute to a tumor-suppressive systemic environment and support emerging evidence that extracellular vesicles act as important mediators of exercise-derived biological effects. In this exploratory pilot study, exercise-responsive enriched L-EVs shifted PC9 molecular programs toward reduced oncogenic signaling, diminished immune-evasion potential, and enhanced apoptotic and cell-cycle inhibitory pathways ─ patterns consistent with the anti-cancer effects reported for exercise-induced small EVs in preclinical models. While causality requires functional validation and individual-level analyses, our results identify L-EVs as a promising and previously underexplored component of exercise-mediated systemic regulation. These observations highlight the importance of studying distinct EV subtypes and underscore the need for future work integrating functional assays, protein-level confirmation, and absolute quantification of EV-miRNAs to refine the mechanistic role of L-EVs. Furthermore, the dynamic responsiveness of circulating EV cargo to exercise in older adults remains an important area for future investigation.

## Supplementary Information

Supplementary Material 1

Supplementary Material 2

## Data Availability

The data that support the findings of this study are not openly available due to reasons of sensitivity and are available from the corresponding author upon reasonable request.

## Abbreviations

EV: Extracellular vesicle
L-EV: Large extracellular vesicle
NTA: Nanoparticle tracking analysis
TEM: Transmission electron microscopy
PFP: Platelet-free plasma
RT-qPCR: Reverse transcription quantitative polymerase chain reaction
miRNA: MicroRNA
DEG: Differentially expressed gene
DEmiR: Differentially expressed microRNA
KEGG: Kyoto Encyclopedia of Genes and Genomes
IPA: Ingenuity Pathway Analysis
EGFR: Epidermal growth factor receptor
NSCLC: Non-small cell lung carcinoma
LUAD: Lung adenocarcinoma
PC9: Human lung adenocarcinoma cell line PC9
GAPDH: Glyceraldehyde 3-phosphate dehydrogenase
