# Supplementary material for: From motion to molecules: exercise-triggered large EVs suppress oncogenic signaling in lung adenocarcinoma cells – an in vitro pilot study
Source: Cancer Cell Int. 2026 Jul 29;26:350. doi: 10.1186/s12935-026-04429-6 (PMC13700146; doi:10.1186/s12935-026-04429-6)
Supplement: Supplementary file 1 [file 12935_2026_4429_MOESM2_ESM.pdf]

Supplementary Data

From Motion to Molecules: Exercise-Triggered Large EVs Suppress Oncogenic Signaling in Lung Adenocarcinoma – An in vitro pilot study

Zoltan Adam[1], Kitti Garai[1*], Abigel Sebok-Tornai [2,3], Marta Wilhelm[4] and Krisztian Kvell[1]

[1] Department of Pharmaceutical Biotechnology, Faculty of Pharmacy, University of Pécs, Pécs, Hungary
[2] Histology and Light Microscopy core facility at the Szentágothai Research Centre of the University of Pécs, Pécs, Hungary
[3] Department of Laboratory Medicine, University of Pécs, Medical School, Pécs, Hungary
[4] Faculty of Science, Institute of Sport Sciences and Physical Education, University of Pécs, Pécs, Hungary
[*]Correspondence: garai.kitti@pte.hu

Table of Contents

From Motion to Molecules: Exercise-Triggered Large EVs Suppress Oncogenic Signaling in Lung Adenocarcinoma – An in vitro pilot study 1
Supplementary: Material and Methods 2
Participants 2
Fig. S1 Flowchart of participant recruitment and selection. 3
Fig. S2 Self-reported frequency of physical exercise among participants (n = 9). 3
Table S1 Self-reported exercise habits and activity types of study participants. 4
Assessment of body composition 4
Single bout endurance exercise test protocols 4
Table S2 Modified Bruce protocol where the slope and the velocity start from a lower workload. 4
Table S3 Applied protocol for the cycle ergometry. 5
Blood sample collection 6
Preparation of serum and plasma 6
Isolation and enrichment of L-EVs 6
Nanoparticle Tracking Analysis (NTA) 6
Cell culture and treatment 7
Fig. S3 Cultured PC9 cells at the start (0h) and after the treatment with L-EVs. 7
Table S4 Adaptation protocol for sequentially increasing Exosome Depleted FBS containing RPMI (ED-RPMI). 7
.... 8
Fig. S4 Effect of exercise-conditioned L-EVs on PC9 cell viability assessed by trypan blue exclusion. . 8
RNA isolation and cDNA synthesis 8
Fig. S5 cel-miR-39 spike-in control across experimental groups. 9
Quantitative (q)RT-PCR 9
Bioinformatic Analysis of miRNA Targets, Genes and Associated Pathways 9
Supplementary: Results 10

EV Characteristics and Exercise Effects 10
Differential miRNA and Gene Remodelling of PC9 Cells by Pre- and Post-Exercise L-EVs 10
Fig. S6 Statistical comparison of L-EV concentration of exercise groups. 10
Table S5 Coverage of lung adenocarcinoma–relevant pathways within the TaqMan® Human Molecular Mechanisms of Cancer Panel 11

Supplementary: Material and Methods

Participants

Participants were excluded from the study if they took prescription drugs with anticoagulant effect or had signs of chronic disease or injury. To ensure that circulating L-EV profiles were not confounded by underlying health conditions ─ and that all participants could safely complete maximal exercise testing ─ comprehensive exclusion criteria were applied. Individuals with chronic diseases known to alter EV secretion or inflammatory status were excluded, including cardiovascular disorders (e.g., ischemic heart disease, arrhythmias, hypertension), metabolic conditions (e.g., diabetes mellitus), respiratory diseases (e.g., COPD, asthma), hepatic or renal impairment, autoimmune diseases, and other chronic inflammatory disorders. These conditions are well documented to influence endothelial-, immune-, or platelet-derived EV release and could bias exercise-induced EV responses. Participants taking medications with known effects on EV secretion, haemostasis, or exercise performance (e.g., anticoagulants, antiplatelet agents, corticosteroids, immunosuppressants, lipid-lowering drugs, β-blockers, or regular NSAID use) were also excluded. For the safety and validity of ergometry testing, individuals with recent injuries (<6 weeks), musculoskeletal pain, joint instability, orthopedic limitations, or neurological conditions affecting balance were excluded, as were those with acute infections, dehydration, anaemia, uncontrolled hypertension, or other factors impairing maximal effort tolerance. Additional exclusion criteria included poor venous access, haematological abnormalities, or bleeding disorders that could interfere with consistent blood sampling. Physical activity levels were assessed using the International Physical Activity Questionnaire (IPAQ) long form and a general lifestyle questionnaire, which also captured alcohol use, smoking status, and detailed exercise habits (type, frequency, duration). Participants were eligible if they were >60 years old, had engaged in regular physical activity for at least 25 years, and met all health and safety criteria to ensure a homogeneous, healthy cohort. Among the included individuals, exercise modalities included running, cycling, swimming, walking, hiking, and spinning; 54% reported exercising daily, and the remaining 46% exercised at least twice weekly. Together, these criteria ensured a well-defined, healthy sample suitable for investigating exercise-induced changes in circulating L-EVs.

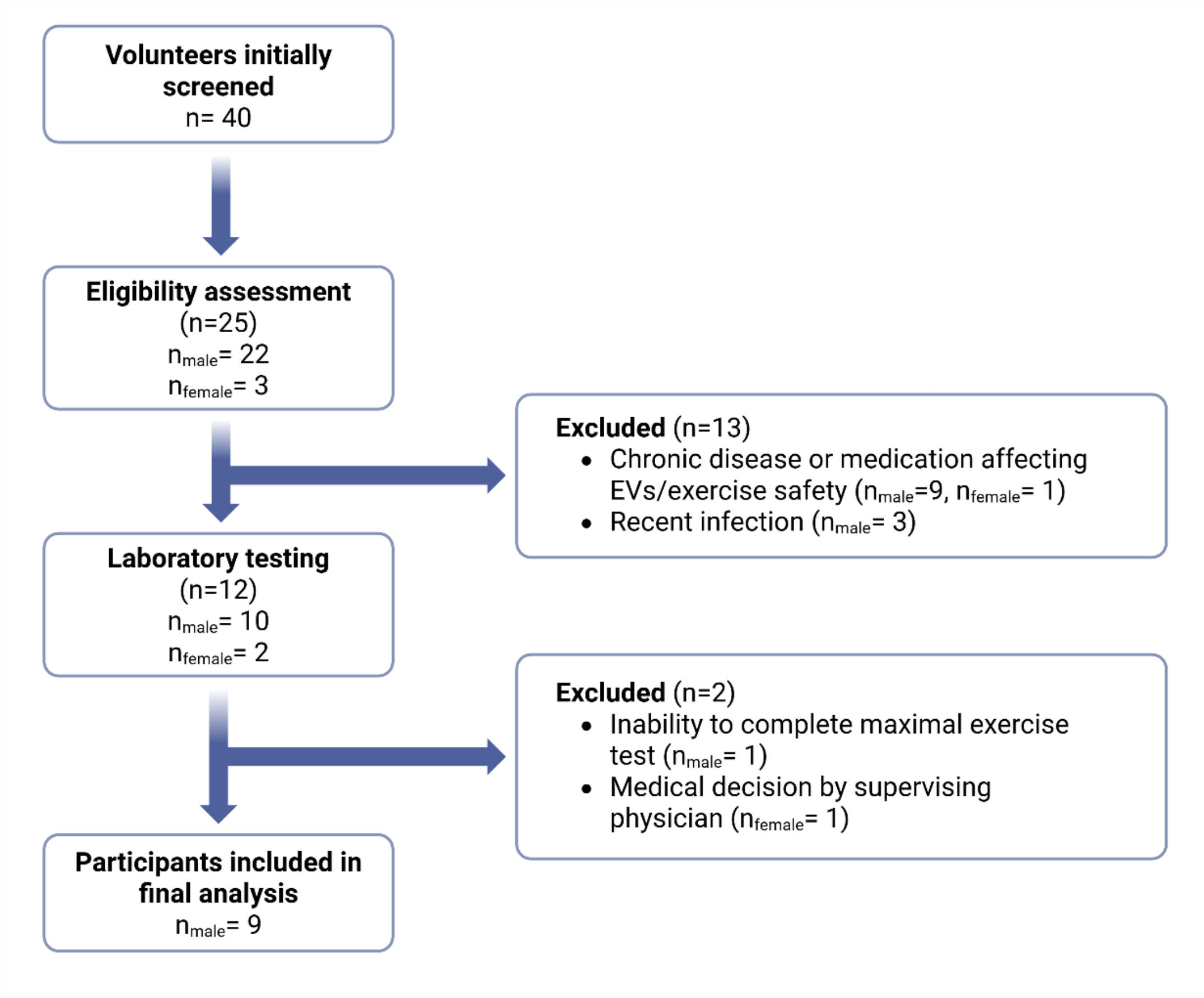

Fig. S1 Flowchart of participant recruitment and selection. Diagram summarizing the recruitment, screening, eligibility assessment, and exclusion of participants in the quasi-experimental human study. Reasons for exclusion included medical conditions, medication use, safety considerations, and failure to meet predefined age or exercise history criteria. Only male participants meeting all inclusion criteria were included in the final analysis.

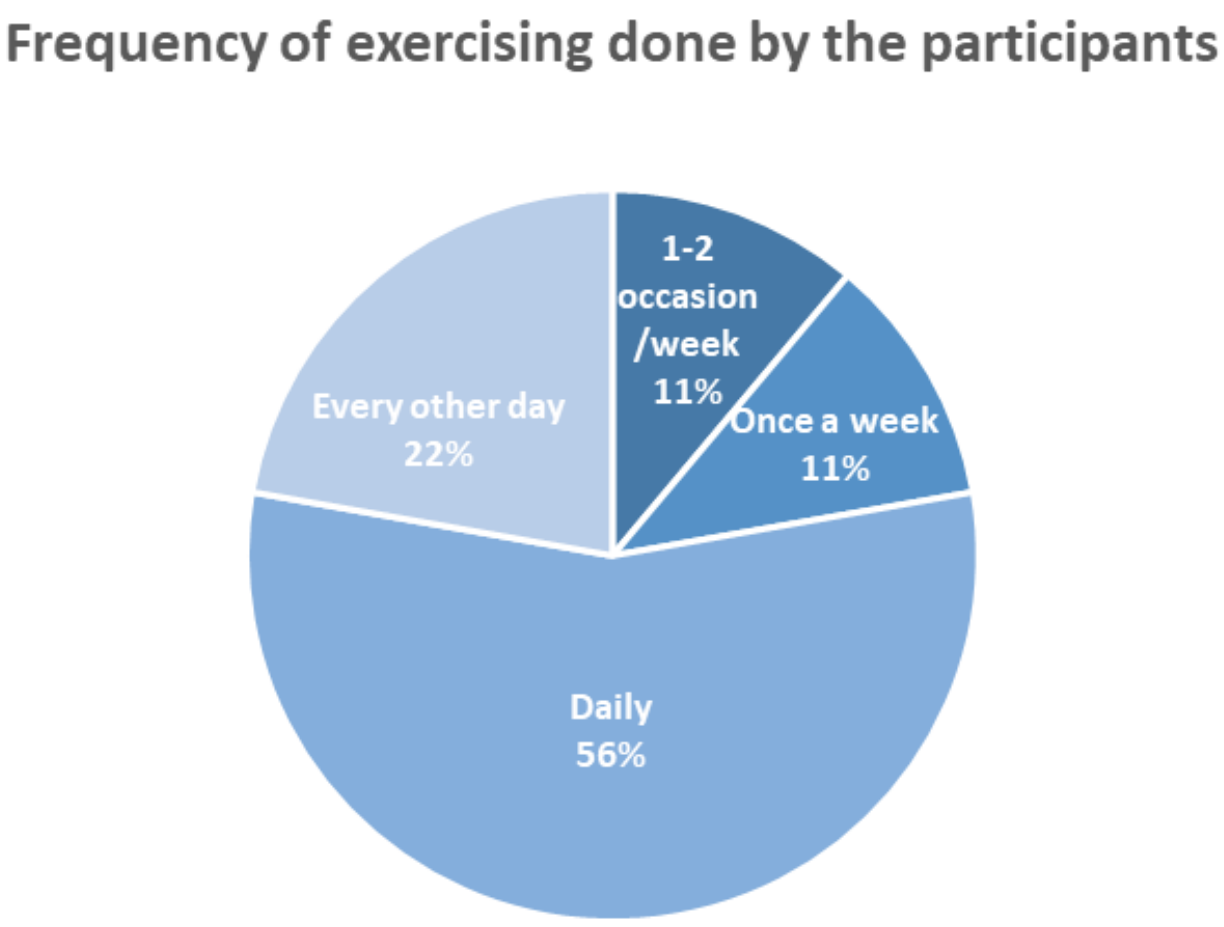

Fig. S2 Self-reported frequency of physical exercise among participants (n = 9). Most participants (56%) reported engaging in daily physical activity, while 22% exercised every other day, and the remaining 22% exercised once or twice per week.

Table S1 Self-reported exercise habits and activity types of study participants. Participants (n = 9) reported engagement in various physical activities, including running, swimming, weightlifting, cycling, spinning, and others. Each activity is marked with a "1" if practiced. The final column indicates the typical frequency of exercise per participant. The bottom row shows the total number of participants engaging in each activity. Most participants reported regular physical activity, with running being the most common (8 out of 9).

Participant	Running	Swimming	Weightlifting	Cycling	Spinning	Skating	Walking	Hiking	Skiing	Adrenaline sports	Martial arts	Frequency of exercising
Participant 1.	1	1	0	1	0	0	0	0	1	0	0	1-2 occasion /week
Participant 2.	1	0	0	0	0	0	0	1	0	0	0	Once a week
Participant 3.	1	1	0	1	0	0	0	0	0	0	0	Daily
Participant 4.	1	1	1	1	0	1	0	1	0	0	0	Daily
Participant 5	1	0	0	0	0	0	1	0	0	0	0	Daily
Participant 6.	1	1	0	1	1	0	0	0	0	0	0	Daily
Participant 7.	0	0	0	0	1	0	0	0	0	0	0	Every other day
Participant 8.	1	1	1	1	0	0	0	0	0	0	0	Daily
Participant 9.	1	0	0	1	0	0	0	1	0	0	0	Every other day

Assessment of body composition

Body height was measured with a Martin type anthropometer. The InBody 770 (Inbody Co., LTD, Seoul, Korea) multi-frequency bioelectrical impedance analyser (BIA) device was used for the body composition measurement. Participants were instructed to stand on the device's platform barefoot with the soles of their feet on the electrodes, then grasped the handles to maintain direct contact with the electrodes. For the duration of the measurement, they were instructed to stand still with their arms fully extended and their shoulder joints abducted to about a 30-degree angle.

Single bout endurance exercise test protocols

All exercise tests were performed as symptom-limited incremental cardiopulmonary exercise tests (CPET) with continuous monitoring of oxygen uptake (VO_2), ventilation, heart rate, and respiratory exchange ratio (RER). Maximal effort was verified by established CPET criteria, including attainment of high RER values (>1.10), near age-predicted maximal heart rate, and plateauing of VO_2 despite increasing workload, ensuring comparable relative exercise intensity across treadmill and cycle ergometer protocols.

The treadmill test: The test was performed on a Full Vision TMX 425 treadmill (FullVision Inc, Newton, KS) connected to a Cardiovit AT-104 PC (Reomed AG, Switzerland). The exercise test was performed following the modified Bruce protocol (Table 1). In this context the slope and the velocity of the ramp increase at a lower workload than the standard test. This modified protocol ensures a progressive improvement of efforts.

Table S2 Modified Bruce protocol where the slope and the velocity start from a lower workload.

Step	Time (min)	Speed (km/h)	Slope (%)
1	3	2.7	10
2	3	4	12

3	3	5.4	14
4	3	6.7	16
5	3	8	18
6	3	8.8	20
7	3	9.6	22
8	3	10.4	24
9	3	11.2	26
10	3	12	28

The cycle ergometer test: Participants performed graded exercise test on an electromagnetically braked cycle ergometer (Schiller CH-6340, BAAR, Switzerland). During the exercise, the cadence should be maintained at a constant rate of 70 repetitions per minute, the first workload was 25 W and increased to 25 W every 1 minute to subjective exhaustion. If the participant could not maintain the speed at the correct value for more than 15 seconds, the test was terminated.

Table S3 Applied protocol for the cycle ergometry.

Step	Time (min)	Increment (W)
1	1	50
2	1	75
3	1	100
4	1	125
5	1	150
6	1	175
7	1	200
8	1	225
9	1	250
10	1	275
11	1	300
12	1	325

Blood sample collection

Procedure

After applying a light tourniquet, we used a 21 Gauge needle to venipuncture an antecubital vein using a closed blood sampling system (BD Vacutainer®). Collection tubes were then gently turned up and down several times to mix the anticoagulant with the drawn sample. The blood collection tubes were held upright at room temperature until centrifugation.

- Blood collection tube specifications:
- EDTA (K3EDTA; 3 mL; art.no.: 368857)
- native additive-free separator tubes (SSTTM II Advance; 8,5 mL; art.no.: 367953)
- sodium fluoride-containing tubes (SF; 2 mL; art.no.: 367921)

All laboratory parameters, including total blood cell counts, plasma lactate, serum albumin, and creatine kinase hsCRP levels were determined by automated routine laboratory techniques at the Department of Laboratory Medicine (reg.no.: NAH-9-0008/2021; University of Pécs, Clinical Centre, Pécs, Hungary).

Blood cell parameters were quantified using the CellDyn 3700 system, a multi-parameter automatic haematology analyser by Abbott Diagnostics. CRP levels were determined using the Cardiac C-Reactive Protein (Latex) High Sensitive turbidimetric immunoassay according to the manufacturer's instructions. Cortisol levels were assessed using the Cortisol RIA Kit (Beckman Coulter, Cat.: IM1841) on the RIA-mat 280 automated analyser (Stratec Gmbh, Birkenfeld, Germany) following the manufacturer's instructions.

Preparation of serum and plasma

- ISTH protocol for platelet-free-plasma (PFP) isolation:

Blood was centrifugated at 2500 x g for 15 minutes at room temperature (RT). After 15 minutes, platelet-poor plasma (PPP) was aspirated and placed in a new 15 mL centrifuge tube (Greiner Bio-One™ CELLSTAR™; art. no.: 188271), leaving at least 500 µL of the supernatant at the bottom of the tube to avoid contamination with cells. The PPP sample was centrifuged at 2500 x g for 15 minutes at RT. PFP was then collected, leaving at least 100 µL at the bottom of the tubes.

Isolation and enrichment of L-EVs

Material specifications:

- PBS without Ca and Mg (Lonza, Walkersville, MD, USA cat. no.: BE17-516Q)
- 800 nm filter (Acrodisc, Pall Corp., Port Washington, NY, USA, PN: 4618)

Nanoparticle Tracking Analysis (NTA)

Settings:

Temperature: 25°C; syringe pump speed: 50; viscosity: 0.889; capture: 10 x 60 seconds. Standardized nanoparticle dilutions from the manufacturer were used to calibrate the instrument for nanoparticle size and quantity measurements before each experimental run. Video acquisitions were performed and

analysed with NTA software v3.4 with the following settings: auto background subtraction/blur/minimum track length. The detection threshold was set according to the sample to reach the best signal-to-noise ratio.

Cell culture and treatment

Material specifications:

- FBS (Gibco; Cat.: 10270-106; Lot.: 2232237; Paisley, UK)
- penicillin/streptomycin (Lonza ref. DE 17–602 E, lot. 5MB068)
- T25 cell culturing flask (TPP, Cat#90026)
- 24-well plate (VWR, Cat# 734-232)

Table S4 Adaptation protocol for sequentially increasing Exosome Depleted FBS containing RPMI (ED-RPMI).

	100% cRPMI	25% EDRPMI	50% EDRPMI	75% EDRPMI	100% EDRPMI
**cRPMI**	100	75	50	25	0
**ED-RPMI**	0	25	50	75	100

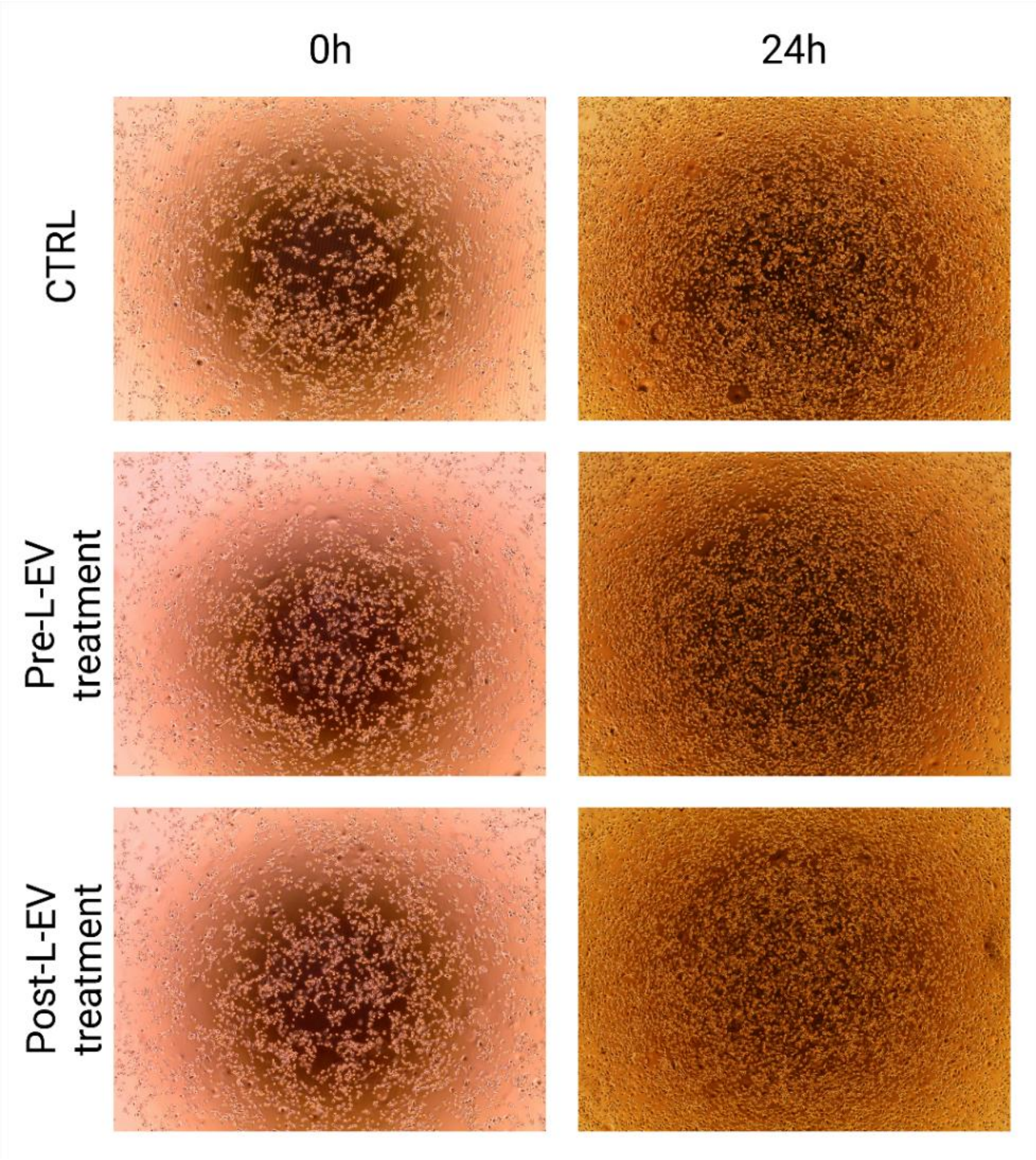

Fig. S3 Cultured PC9 cells at the start (0h) and after the treatment with L-EVs.

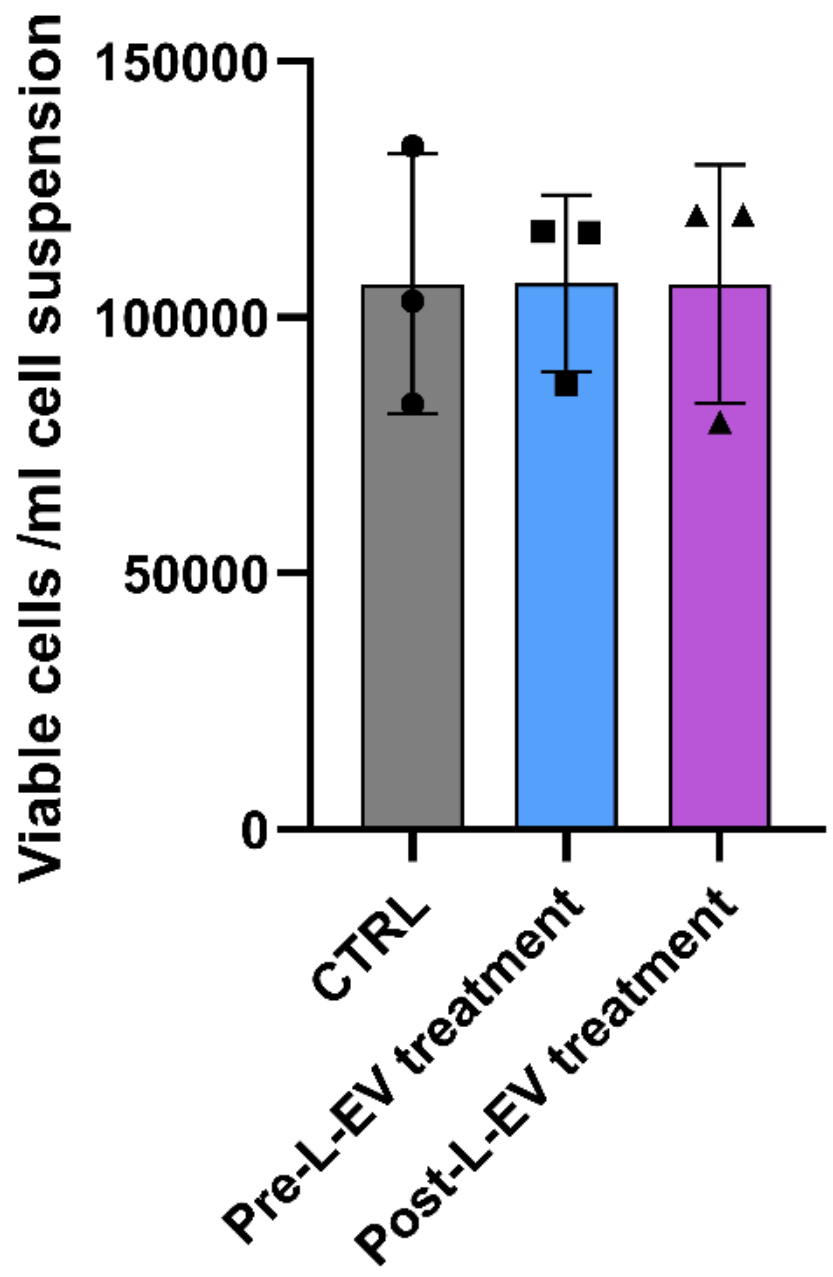

Fig. S4 Effect of exercise-conditioned L-EVs on PC9 cell viability assessed by trypan blue exclusion. PC9 cells were treated for 24 h with pooled Pre- and Post-exercise L-EV–enriched fractions or left untreated (CTRL). Viable cells were quantified by trypan blue exclusion and expressed as cells/mL of suspension. Bars represent mean ± SD from three independent biological replicates, with individual data points overlaid. No statistically significant differences in viable cell counts were observed between conditions, indicating that L-EV treatment did not markedly affect cell number under these conditions ($p<0.05$).

RNA isolation and cDNA synthesis

- Total Exosome RNA & Protein Isolation Kit (Invitrogen, Cat# 4478545)
- mirVana™ miRNA Isolation Kit (Invitrogen, Cat# AM1561)
- TaqMan™ Advanced miRNA cDNA Synthesis Kit (Cat# A25576, Applied Biosystems, protocol: publication number MAN0016122)
- High-Capacity cDNA Reverse Transcription Kit (Cat# 4368814)

- cel-miR-39 spike-in oligo: Phos-UCACCGGGUGUAAAUCAGCUUG (Invitrogen, Cat# 10620310)

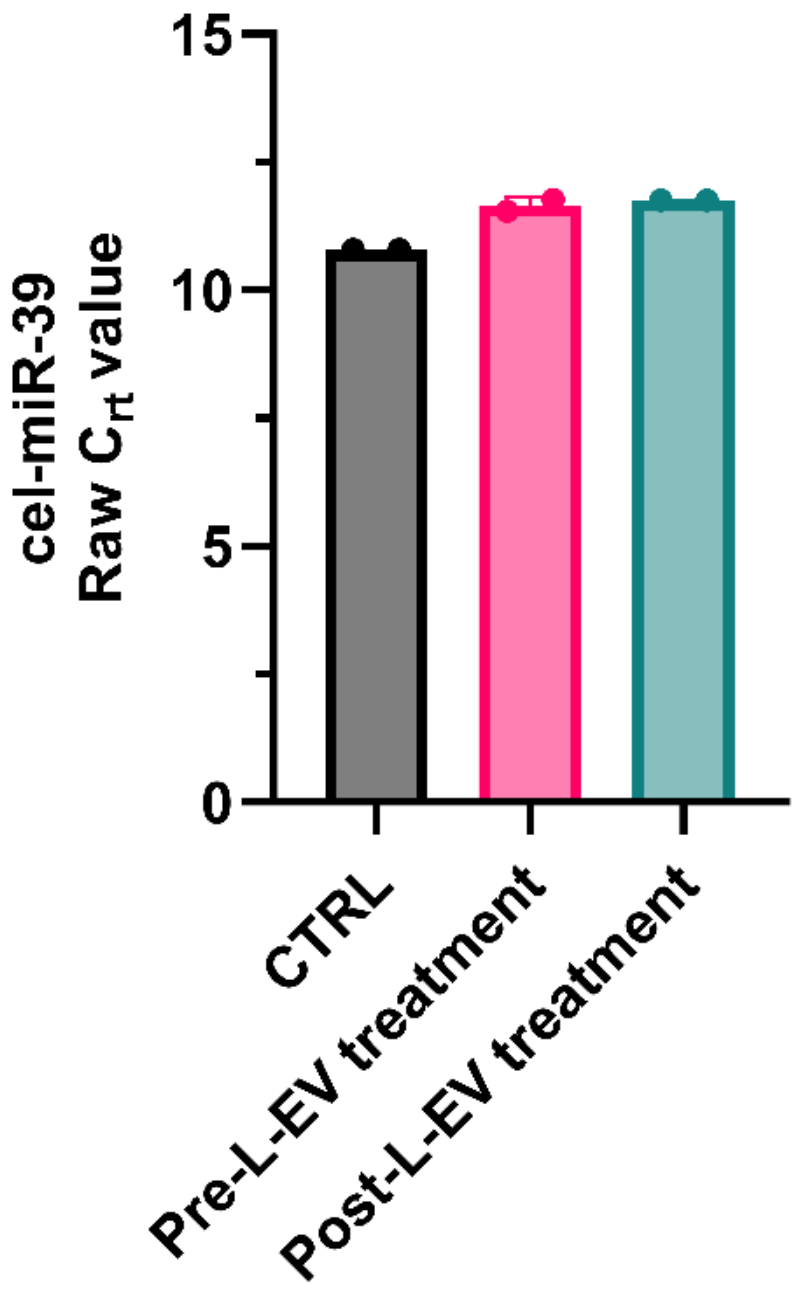

Fig. S5 cel-miR-39 spike-in control across experimental groups. Crt values (mean ± SD of duplicates) demonstrate uniform RNA isolation and miRNA RT-qPCR efficiency across CTRL, Pre-L-EV and Post-L-EV treatment samples.

Quantitative (q)RT-PCR

- TaqMan® Advanced miRNA Human A cards, Applied Biosystems

A 1:10 dilution in 0.1X TE buffer (Thermo Fisher Scientific, Cat No. AM9849) of pre-amplified cDNA, along with TaqMan® Fast Advanced Master Mix was loaded into each card. The cards were run on a QuantStudio 12k Flex Real-Time PCR System (Life Technologies) with the amplification protocol of the corresponding user guide (MAN0016122 Rev. B.0). Data quality was evaluated based on the following criteria: RT-PCR products were omitted (1) if the amplification score < 1.0 with an irregular amplification curve; (2) if the amplification score was reported as 'inconclusive' or 'undetected' (3) Cq confidence < 0.8 (4) Crt> 32.

- TaqMan Array Human Molecular Mechanisms of Cancer (assay nº 4418806, Applied Biosystems)

Bioinformatic Analysis of miRNA Targets, Genes and Associated Pathways

- MiRTarBase (available online at http://mirtarbase.mbc.nctu.edu.tw/php/index.php)
- DIANA-mirPath v4.0 (available online: https://diana-lab.e-ce.uth.gr/app/miRPathv4)

Lists of significantly altered DEGs and DEmiRs from each treatment condition were uploaded into IPA. Core analysis was performed using default parameters, incorporating both observed and highly predicted confidence level.

Supplementary: Results

EV Characteristics and Exercise Effects

Differential miRNA and Gene Remodelling of PC9 Cells by Pre- and Post-Exercise L-EVs

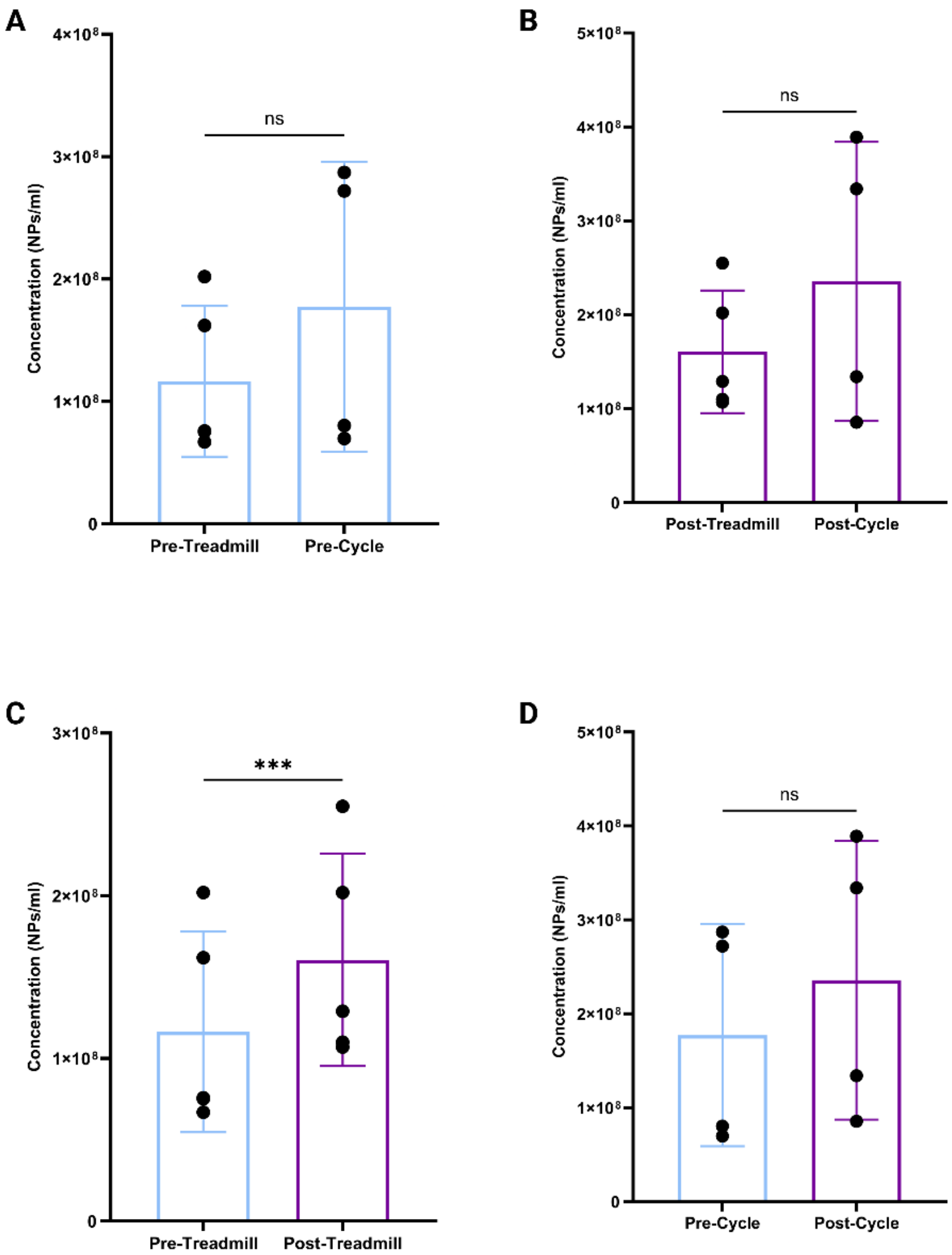

Fig. S6 Statistical comparison of L-EV concentration of exercise groups. (**A**) Between group pre-exercise L-EV concentration comparison of the two exercise modality groups. (**B**) Between group post-exercise L-EV concentration comparison of the two exercise modality groups. (**C**) Within group pre- and post-exercise L-EV concentration comparison of participants completed the treadmill protocol. (**D**) Within group pre- and post-exercise L-EV concentration comparison of participants completed the cycle ergometry protocol. Ns: non-significant, *** p< 0.001

Table S5 Coverage of lung adenocarcinoma–relevant pathways within the TaqMan® Human Molecular Mechanisms of Cancer Panel

Signaling pathway	Representative genes included in the panel	Differentially expressed genes observed in this study	Relevance to NSCLC / PC9 cells
**EGFR / ERBB signaling**	EGFR, ERBB2, GRB2, SHC1, SOS1	ELK1 ↓, SHC1 ↑ (Pre)	Central driver pathway in EGFR-mutant NSCLC and PC9 cells; regulates proliferation, survival, and TKI sensitivity
**PI3K–AKT signaling**	AKT1, AKT2, PIK3CA, PIK3R1, PTEN	–	Core survival pathway downstream of EGFR; alterations contribute to therapeutic resistance
**MAPK signaling**	MAPK1, MAPK3, MAPK8, MAPK14, RAF1, MAP2K1	ELK1 ↓	Controls proliferation, stress signaling, and metastasis in lung adenocarcinoma
**MYC/MAX transcriptional axis**	MYC, MAX	MAX ↓ (Post)	MYC/MAX heterodimers drive oncogenic transcription, metabolic adaptation, and immune evasion
**Cell cycle regulation**	CCND1, CCND2, CCND3, CDK2, CDK4, CDKN1A, CDKN2B	CCND2 ↑ (Pre), CDKN2B ↑ (Post)	Dysregulated cell cycle progression is a hallmark of NSCLC
**Apoptosis signaling**	BAX, BCL2, BID, CASP8, CASP9, FADD	CASP9 ↑, FADD ↑ (Post)	Induction of apoptosis is a key tumor-suppressive mechanism
**Immune evasion / death receptor signaling**	FAS, FASLG, TNFSF-associated genes	FASLG ↓ (Post)	Fas–FasL signaling contributes to immune escape in lung cancer
**WNT / β-catenin signaling**	CTNNB1, LEF1, WNT1, FZD1, DVL1	LEF1 ↑ (Pre)	Promotes proliferation and EMT in NSCLC
**TGF-β signaling**	TGFB1, TGFBR1, TGFBR2, SMAD4	–	Involved in tumor progression, EMT, and immune modulation
**NF-κB signaling**	NFKB1, NFKB2, RELA, NFKBIA	–	Regulates inflammation, survival, and resistance mechanisms
**Angiogenesis**	VEGFA, KDR	–	Supports tumor vascularization and growth
**Adhesion / migration**	FN1, ITGAV, ITGB1, ITGB3, RAC1, RHOA	–	Contributes to invasion and metastatic potential

All listed genes were quantitatively assessed in the present study. Only genes meeting predefined differential expression criteria are reported as altered, reflecting selective modulation of key regulatory nodes rather than global transcriptional activation. Arrows (↑/↓) indicate upregulation or downregulation, respectively. “—” indicates no differential expression change, Pre: measured in Pre-exercise L-EV treated PC9 cells, Post: measured in Post-exercise L-EV treated PC9 cells
